# MITE infestation accommodated by genome editing in the germline genome of the ciliate *Blepharisma*

**DOI:** 10.1073/pnas.2213985120

**Published:** 2023-01-20

**Authors:** Brandon K.B. Seah, Minakshi Singh, Christiane Emmerich, Aditi Singh, Christian Woehle, Bruno Huettel, Adam Byerly, Naomi A. Stover, Mayumi Sugiura, Terue Harumoto, Estienne C. Swart

**Affiliations:** ^a^Max Planck Institute for Biology, Tuebingen 72072, Germany; ^b^Max Planck Genome Center Cologne, Max Planck Institute for Plant Breeding, Cologne 50829, Germany; ^c^Department of Computer Science and Information Systems, Bradley University, Peoria, IL 61625; ^d^Department of Biology, Bradley University, Peoria, IL 61625; ^e^Department of Chemistry, Biology, and Environmental Sciences, Faculty of Science, Nara Women’s University, Nara 630-8506, Japan

**Keywords:** DNA elimination, mobile element, selfish gene, micronucleus, macronucleus

## Abstract

During their development following sexual conjugation, ciliates excise numerous internal eliminated sequences (IESs) from a copy of the germline genome to produce the functional somatic genome. Most IESs are thought to have originated from transposons, but the presumed homology is often obscured by sequence decay. To obtain more representative perspectives on the nature of IESs and ciliate genome editing, we assembled 40,000 IESs of *Blepharisma stoltei*, a species belonging to a lineage (Heterotrichea) that diverged early from those of the intensively studied model ciliate species. About a quarter of IESs were short (<115 bp), largely nonrepetitive, and with a pronounced ~10 bp periodicity in length; the remainder were longer (up to 7 kbp) and nonperiodic and contained abundant interspersed repeats. Contrary to the expectation from current models, the assembled *Blepharisma* germline genome encodes few transposases. Instead, its most abundant repeat (8,000 copies) is a Miniature Inverted-repeat Transposable Element (MITE), apparently a deletion derivative of a germline-limited Pogo-family transposon. We hypothesize that MITEs are an important source of IESs whose proliferation is eventually self-limiting and that rather than defending the germline genomes against mobile elements, transposase domestication actually facilitates the accumulation of junk DNA.

## Significance

Ciliates are microbial eukaryotes with a unique life cycle: their cells contain two kinds of nuclei, and during development, they eliminate thousands of DNA segments thought to have originated from transposons. Because there are significant differences in DNA elimination between the two best-studied ciliate groups, we sequenced the DNA destined for elimination in *Blepharisma*, a distantly related species whose phylogenetic position allowed us to evaluate which aspects of the elimination are probably ancestral, including properties of the eliminated DNA and of small RNAs that may target them. We hypothesize that truncated transposon derivatives called MITEs, which are particularly abundant in the eliminated DNA, not only are abundant sources of this DNA but also contribute to retarding its generation.

Ciliates are microbial eukaryotes that maintain separate germline and somatic genomes in each cell, housed in two distinct types of nuclei. During the sexual life cycle, the germline micronuclei (MICs) give rise to new somatic macronuclei (MACs) via a process of small RNA (sRNA)-assisted DNA elimination and DNA amplification; the MACs are then the sites of most gene expression in vegetative cells. Genome segments limited to the germline, called internal eliminated sequences (IESs), are excised during development from MIC to MAC, so that the MAC genome content is a subset of that of the MIC. Each of the few ciliate taxa studied intensively to date has its own peculiarities. For example, typical IESs in *Paramecium* are short, have unique sequence content, and are precisely excised, whereas IESs in *Tetrahymena* are longer, more repetitive, and imprecisely excised (1–3).

**Fig. 1.**
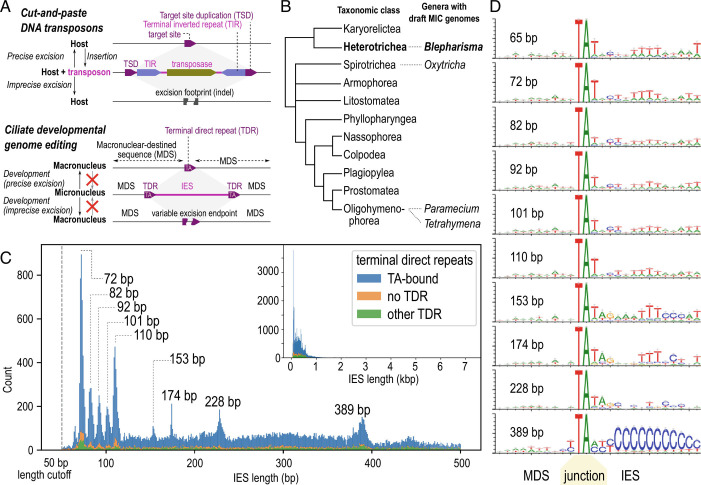
A “hybrid” IES landscape with periodic length peaks for short IESs. (*A*) Comparison of cut-and-paste DNA transposons (*Above*) and ciliate genome editing (*Below*), showing parallels between TSD of transposons and TDRs bounding IESs, and effects of precise vs. imprecise excision. (*B*) Genera with draft MIC genomes relative to diagrammatic tree of ciliate classes (following ref. 14), branch lengths arbitrary. (*C*) IES length histogram (0 to 500 bp (*Inset*: full range), stacked bars for types of TDRs at IES boundaries. Peaks for IES size classes discussed are marked. (*D*) Sequence logos for MDS-IES junctions for TA-bound IESs of specific size classes, centered on the “TA”. See also *SI Appendix*, Figs. S1–S3.

Ciliate IESs are thought to have originated from cut-and-paste DNA transposons (4) (Fig. 1*A*) because i) the 5′-TA-3′ motifs of IES boundaries in *Paramecium* and *Euplotes* resemble the terminal direct repeats (TDRs) of Tc1/Mariner-superfamily transposons (5); ii) transposon-derived “domesticated” excisases are used to remove IESs (6–8); and iii) intact transposons encoding transposases are mostly germline-limited (2, 9–11). Recently, IESs with nonautonomous mobile elements resembling Miniature Inverted-repeat Transposable Elements (MITEs) have been reported in *Paramecium* (12). MITEs are deletion derivatives of Tc1/Mariner transposons that are common in eukaryotes and bacteria (13) and are generally short (<500 bp), without coding sequences, and bounded by terminal repeats. However, the autonomous counterparts of most putative *Paramecium* MITEs, including the most abundant ones with thousands of copies, have not been identified.

Developmental DNA elimination in ciliates has been viewed as “genome defense” because the process removes IESs, which not only derive from selfish genetic elements (transposons) but are often intragenic and hence deleterious if not removed (15). This view was popularized in part because of parallels to other eukaryotes, in which small-RNA-mediated DNA heterochromatinization is thought to suppress the proliferation of mobile elements (16–18). Ciliates have also been hypothesized to use development-specific sRNAs to guide DNA elimination. For example, in the class Oligohymenophorea (e.g., *Tetrahymena*, *Paramecium*), they appear to mark sequences for elimination (15, 19, 20), whereas in class Spirotrichea (e.g., *Oxytricha*) they appear to mark sequences to be retained (21, 22). Histone modifications are also required for elimination (23, 24). sRNAs may not always be strictly necessary. For example, in *Paramecium*, knockdown of key sRNA biogenesis enzymes had a smaller effect on shorter IESs than on longer ones and was only weakly correlated with the more potent effects of knocking down the main IES excisase (20, 25).

Other phenomena during genome editing differ markedly among the few model species that have been studied in detail [reviews: (18, 26, 27)]. For example, in all species, germline chromosomes are fragmented to some degree into smaller, somatic ones; in most species, somatic chromosomes contain hundreds to thousands of genes, but spirotrichs produce extremely short somatic “nanochromosomes” with only one or a few genes. Similarly, “unscrambling” of nonsequential MAC-destined sequences into the correct order in the somatic genome occurs frequently in some spirotrichs [e.g., *Oxytricha* and *Stylonychia* (28)] and infrequently in *Tetrahymena* (1) and has not been reported in *Paramecium* or other ciliates. Further evaluation of these issues is hampered by the paucity of draft-quality germline-genome sequences, which are available for only two of 11 class-level taxa (following the taxonomy of ref. 14), Oligohymenophorea (1, 2, 12, 29) and Spirotrichea (30) (Fig. 1*B*).

Because it is not yet clear which genome-editing elements are common to all ciliates, we studied *Blepharisma stoltei* (class Heterotrichea), a species whose last common ancestor with other ciliates whose germline genomes have been sequenced is the last common ancestor of all ciliates (31). *Blepharisma* has been a laboratory model for photobiology (32) and for mating type recognition through diffusible mating factors (gamones) (33–36), so cultivated strains and protocols for inducing conjugation and development are available. An accurate, highly contiguous draft sequence of the somatic genome is now also available (37). The somatic genome encodes a probable IES excisase, *Blepharisma* PiggyMac (BPgm), a PiggyBac-family homolog that is most closely related to the main IES excisases of *Paramecium* (PiggyMac) and *Tetrahymena* (Tpb2). Other somatic PiggyBac paralogs are also present but lack the complete “catalytic triad” of the classical PiggyBac transposase from cabbage looper moths that is necessary for excisase activity, similar to the situation in *Paramecium* (38). BPgm is upregulated during the formation of the new MAC together with other development-specific genes, including homologs of sRNA biogenesis proteins implicated in genome editing (37).

In this study, we assembled a draft sequence of the *Blepharisma stoltei* germline genome, to identify genome editing characteristics that are likely to have been present in the ciliate last common ancestor. Through single-molecule long-read sequencing and targeted assembly, we could assemble IESs with long, repetitive elements, which would not have been feasible with short-read shotgun sequencing alone. Complementing the genomic analyses, we also sequenced sRNAs expressed during sexual development to find homologs of the scnRNAs that guide DNA elimination in other ciliates.

## Results

### Detection and Targeted Assembly of ca. Forty Thousand Germline-Limited IESs.

To investigate the *Blepharisma stoltei* (hereafter *Blepharisma*) germline genome, we enriched germline MICs from strain ATCC 30299 and reconstructed 39,799 IESs (13.2 Mbp total, average coverage ~45×) scaffolded on the previously assembled 41 Mbp somatic genome (37). We applied a mapping and targeted assembly approach developed for PacBio long reads (39), which could better assemble repetitive elements compared to using short read sequencing (*SI Appendix*, *SI Results* “IES assembly from short vs. long reads”). The reconstructed IESs are limited to those flanked by a MAC-destined sequence pair. About 20% of the library was of such a MIC origin (*SI Appendix*, *SI Results* “MIC sequence coverage and telomeric content”). This MAC-scaffolded germline assembly is here referred to as the “MAC + IES” assembly. About 70% of all predicted IESs were intragenic (within coding sequences or introns), implying that IESs are precisely excised, as they would otherwise cause deleterious translation frameshifts. Given that genes occupied 77% of the somatic assembly (excluding telomeres), there was a small but statistically significant (*P* = 3 × 10^−269^) relative depletion of intragenic IESs.

### A “Hybrid” IES Landscape with Periodic Length Peaks for Short IESs.

Most *Blepharisma* IESs were short (median 255 bp, mean 331 bp), but the distribution was long-tailed (90th percentile 603 bp, max 7251 bp). The length distribution was not unimodal but had multiple peaks at specific length values (Fig. 1*C* and *SI Appendix*, Table S1). It appeared to be a “hybrid” distribution composed of two ranges: a “periodic” range, from ~65 to 115 bp (10,778 IESs) and a “nonperiodic” range, >115 bp (29,021 IESs).

The periodic IES size range contained sharp peaks every 10 to 11 bp, similar to the periodicity of *Paramecium tetraurelia* IESs (2, 29). The first peak in *Blepharisma* was centered at 65 bp, compared to 28 bp in *P. tetraurelia*, and there was no “forbidden” peak, unlike *P. tetraurelia* where an expected second peak at ~38 bp is largely absent (*SI Appendix*, *SI Results “Periodic IES length distribution”*). The most abundant periodic length peaks in *Blepharisma* were at 72 bp and 110 bp. The nonperiodic range (≥115 bp) contained isolated peaks at 153, 174, 228, and 389 bp, which has no obvious periodicity. Only 9,701 IESs (total 1.36 Mbp) were contained within the size classes represented by the above peaks (both periodic and nonperiodic) (*SI Appendix*, Table S1), meaning that most IESs had lengths outside the peak values.

### IESs are Bounded by Heterogeneous Direct and Inverted Terminal Repeats.

In other ciliates, IES boundaries often have conserved terminal repeat motifs that could reflect excisase cut site preferences or IES origins from specific classes of transposons (4). We found heterogeneous direct and inverted repeats at the termini of *Blepharisma* IESs, often correlated to IES size classes, that suggested that they belonged to different families of elements.

About three-quarters of IESs (30,212 IESs, 9.43 Mbp) were bounded by TDRs that contained the subsequence TA (“TA-bound”). Other non-TA TDRs accounted for another 6,566 IESs (2.85 Mbp); the remainder were not TDR-bound, though some may represent assembly errors (Fig. 1*C*). Like most ciliates, *Blepharisma* genomes were AT-rich (somatic 33.5% GC, IESs 33.3% GC), but the number of TA- and TDR-bound sequences was unlikely to be due to nucleotide composition alone (*SI Appendix*, Fig. S2 *A* and *B*). The most common TDRs were simple alternations of T and A (TA, TAT/ATA, TATA), especially in IESs up to 228 bp (*SI Appendix*, Fig. S2*C*), with the exception of TAA/TTA (see below). These motifs likely represent cut site preferences of the excisase because they were also found in MDS sequences that were erroneously excised at a low background frequency (*SI Appendix*, *SI Results* “Cryptic IESs in the MAC genome”).

Terminal inverted repeats (TIRs) at IES junctions were heterogeneous among IES size classes (Fig. 1*D* and *SI Appendix*, Fig. S2*F*), with no single TIR motif conserved across all *Blepharisma* IESs, unlike the common 5′-TAYNR-3′ motif in *Paramecium* (*SI Appendix*, *SI Results* “TIRs and palindromic IESs”). Despite this heterogeneity, TIRs were common and longer than expected by chance, with distinct TIRs associated with specific IES length classes (*SI Appendix*, Fig. S2 *D* and *E*). IESs in the ~389 bp size peak in particular had distinctive direct (TAA/TTA) and inverted repeats, suggesting that they constitute a family of homologous IESs (see “*Pogo/Tigger-Family Transposon with Abundant MITEs*”).

### Repeat Elements Are Abundant in Long, Nonperiodic IESs.

**Fig. 2.**
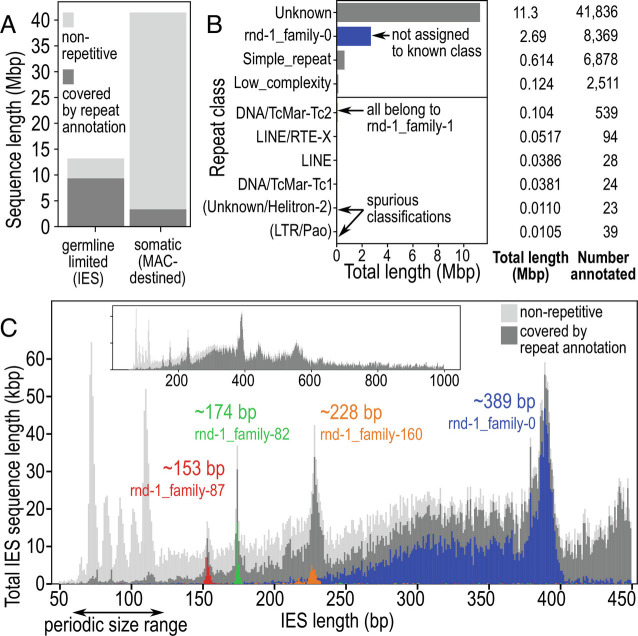
Repeat elements are abundant in long, nonperiodic IESs. (*A*) Total sequence length annotated as interspersed repeats vs. nonrepetitive, in germline-limited vs. somatic parts of the genome. (*B*) Classification of repeat families by RepeatClassifier, and total annotated length per repeat class. (*C*) Total sequence length (vertical axis) per IES size class (horizontal axis), stacked plot of nonrepetitive fraction vs. interspersed repeats, with the most abundant repeat families in the four nonperiodic peaks overlaid in color. *Inset*: Distribution to 1,000 bp. See also SI Appendix, Fig. S4.

Mobile elements that have recently proliferated should appear as interspersed repeat elements in the genome. A quarter of the MAC + IES assembly (12.7 Mbp, 23.3%) was composed of identifiable interspersed repeats; like in other model ciliates (1, 30), they made up a greater proportion of germline-limited IESs (71.0%) than the somatic genome (8.12%) (Fig. 2*A*). The majority of sequence content in longer IESs ≥115 bp was annotated as repetitive, whereas the converse was true for the shorter, periodic IESs (Fig. 2*C*), paralleling *Paramecium*’s short IESs, which are mostly unique sequences (2).

Most interspersed repeats could not be classified into a known transposable element class by RepeatClassifier (Fig. 2*B* and *SI Appendix*, Table S2). The most abundant classifiable type was “DNA/TcMar-Tc2”, all of which actually belonged to a single repeat family rnd-1_family-1, followed by “LINE/RTE-X”. The most abundant family, rnd-1_family-0, was unclassified and made up 21.2% (2.69 Mbp) of total repeats. Families rnd-1_family-0 and rnd-1_family-1 were related and are discussed further below (“*Pogo/Tigger-Family Transposon with Abundant MITEs*”).

Three nonperiodic IES length peaks (153, 174, and 389 bp) could be attributed to specific repeat families, suggesting that they proliferated recently (Fig. 2*C* and *SI Appendix*, Fig. S4*B* and Table S3). This was most pronounced for the ~389 bp peak, where 68.5% of the sequence content belonged to rnd-1_family-0, whereas about a quarter of the ~153 and ~174 bp peaks was composed of repeat families rnd-1_family-87 (palindromic) and rnd-1_family-82, respectively.

### Germline-Limited Repeats Include Transposons with Abundant Nonautonomous MITEs.

**Fig. 3.**
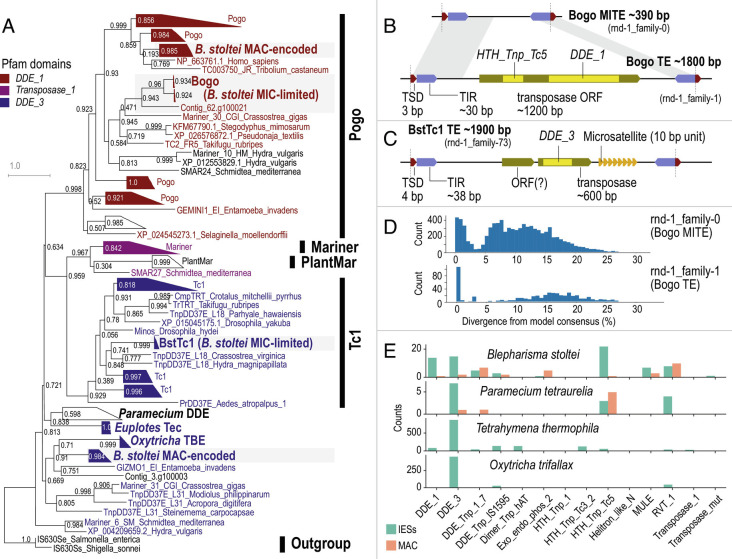
Germline-limited repeats include few autonomous transposons but many MITEs. (*A*) Phylogenetic tree of DDE/D domains for Tc1/Mariner superfamily, including *Blepharisma stoltei* germline-limited (Bogo and BstTc1) and somatic transposases. (*B*) Diagram of features in Bogo and BogoMITE; TSD, TIR, HTH_Tnp_Tc5, DDE_1–conserved domains. (*C*) Diagram of features in BstTc1: DDE_3—conserved domain. (*D*) Histograms of sequence divergence from repeat family consensus for copies of the Bogo and BogoMITE repeat families annotated by RepeatMasker; for rnd-1_family-1, most low-divergence copies (<5% divergence) were short fragments, but all full-length copies were low-divergence. (*E*) Counts of transposase-related domains in different ciliates from six-frame translations of somatic vs. germline-limited genome sequence. See also *SI Appendix*, Figs. S5 and S6.

Unlike *Tetrahymena* and *Oxytricha* where transposases are abundant in the germline-limited IESs but rare in the somatic genome (1, 30), only a few dozen transposase domains were identifiable in either the germline-limited or somatic genomes of *Blepharisma*. Cut-and-paste DNA transposase domains of the DDE/D superfamily identified in *Blepharisma* included DDE_1 and DDE_3 (Tc1/Mariner family), DDE_Tnp_1_7 (PiggyBac), DDE_Tnp_IS1595 (Merlin), and MULE (Mutator) (Fig. 3*E* and *SI Appendix*, Table S4). Not all copies of DDE/D transposase domains in *Blepharisma* contained an intact catalytic triad (*SI Appendix*, *SI Results “Catalytic triad in DDE/D-superfamily transposases”*), suggesting that some may be inactive fragments or pseudogenes. Nonetheless, domains with an intact triad were found in both germline-limited and somatic sequences. In general, the expression level of somatic transposase genes was substantially higher than germline-limited ones (*SI Appendix*, Fig. S6). This contrasts with observations in *Oxytricha*, where germline-limited transposase genes had abundant expression (30).

To identify intact transposon units, we examined the seven repeat families in the MAC + IES assembly that were classified by RepeatClassifier (Fig. 2*B*). Of these, only two were predominantly germline-limited and represented by more than one full-length copy, namely rnd-1_family-1 and rnd-1_family-73 (*SI Appendix*, Table S5). They contained transposases distinct from those found in the MAC genome (Fig. 3 *A*–*C*).

### Pogo/Tigger-Family Transposon with Abundant MITEs.

Repeat elements of rnd-1_family-1 were bound by a ~30 bp TIR 5′-CTC CCC CCC CCC CTC CGT GAG CGA ACA AAA-3′ whose poly-C run length was variable, possibly from assembly errors, and were flanked by a putative target site duplication (TSD) 5′-TAA-3′ (or its reverse complement 5′-TTA-3′) (Figs. 1*D* and 3*B*). All thirty intact (≥95% of consensus length) copies of this family were found within IESs and had high sequence identity to each other (median 0.5% divergence from consensus).

The encoded transposase contained two domains characteristic of the Pogo family in the Tc1/Mariner superfamily: a DDE/D superfamily endonuclease domain (Pfam domain DDE_1) and a helix-turn-helix domain (Pfam domain HTH_Tnp_Tc5) (40). The conserved acidic residues (“catalytic triad”) characteristic of DDE/D transposases (41) were also present, with the motif DD35D, i.e., all three residues were Asp, with 35 residues between the second and third conserved Asp. A phylogeny of the DDE_1 domain placed the transposase in the Pogo/Tigger family, most closely related to the Tc2 subfamily and a sequence from the oyster *Crassostrea*, all of which also had the DD35D motif (Fig. 3*A*). The transposase appeared to be germline-limited, with only 10 partial Tblastn hits in the MAC genome (seven of which were on low-quality “cruft” contigs) that mostly overlapped the HTH_Tnp_Tc5 domain (17 to 84 a.a., E-values 2.3 × 10^−12^ to 1.4 × 10^−6^) and that lacked matches to the DDE_1 domain. However, the TIR did not match previously characterized TIR signatures for the Tc2, Fot, and Pogo subfamilies. A search of all *Blepharisma* IES sequences against HMMs for known DNA transposon TIRs in the Dfam database found only three matches with E-value < 0.01, none from the above subfamilies.

The same TIR and TSD were also found in another repeat family rnd-1_family-0 (*SI Appendix*, Fig. S5), which was the most abundant repeat in the genome (*SI Appendix*, Fig. S4 *A* and *B*); however, members of this family were short elements without any predicted coding sequences. rnd-1_family-0 elements often constituted most of the ~389 bp IES size class (Fig. 2*C*): the TSDs bounding the repeats (TAA/TTA) were the TDRs for most of these IESs (*SI Appendix*, Fig. S2*C*), and the C-rich TIR motif corresponded to the C-rich IES junctions (Fig. 1*D* and *SI Appendix*, Fig. S2*F*). Copies of rnd-1_family-0 were also found nested in longer IESs, suggesting recent proliferation (*SI Appendix*, Fig. S4*C*). Degenerated or partial copies were found in shorter IESs (Fig. 2*C*), with copies >5% divergence from consensus having median length 308 bp vs. 388 bp for copies <5% divergence (Fig. 3*D*).

Therefore, we interpreted rnd-1_family-1 as a new Pogo/Tigger transposon, with a nonautonomous derivative MITE, rnd-1_family-0. We propose the names Bogo for the transposon and BogoMITE for its MITE, as well as the new term “MITIES” (Miniature Inverted-repeat Transposable Internal Eliminated Sequences) to reflect their dual nature as MITEs and IESs. Given their palindromic nature, sequences underlying rnd-1_family-87 and rnd-1_family-160 repeats may also be MITIES.

### Tc1-Family Transposon with Microsatellites.

Another IES-limited repeat family, rnd-1_family-73, also contained a DDE/D-type transposase coding sequence. Twenty-two copies were >80% of the consensus length with low sequence divergence (median 0.6% vs. consensus). A putative complete transposon bounded by a TSD 5′-TATA-3′ and a 38 bp TIR 5′-GTA CCC CCC CCC TCG TTT GTC GCA TTT TCT AGT TTT TT-3′ could be defined after manual curation of repeat boundaries (Fig. 3*C*). Nine of these were mobile IESs, where the TSDs of the transposon also corresponded to the IES junctions. The remaining cases were nested in larger IESs alongside other repeat elements. Ten repeats also contained a microsatellite with ~5 to 42 copies of its 10 bp repeat unit 5′-GGG AAG GAC T-3′ (Fig. 3*C*) not found elsewhere in the genome. We propose the name BstTc1 for this putative transposon.

The transposase encoded in full-length copies of BstTc1 contained a conserved DDE/D superfamily domain DDE_3, phylogenetically affiliated to the Tc1 family although the exact placement is unclear, grouping with only moderate support with Tc1 elements from *Crassostrea* and *Hydra* (Fig. 3*A*). Its catalytic triad motif DD34E differed from previously reported motifs for the Tc1 family, DD41D, DD37D or DD36E (42), so it may be a novel subfamily.

### Non-LTR Retrotransposon Sequences in Both the Somatic and Germline Genomes.

**Fig. 4.**
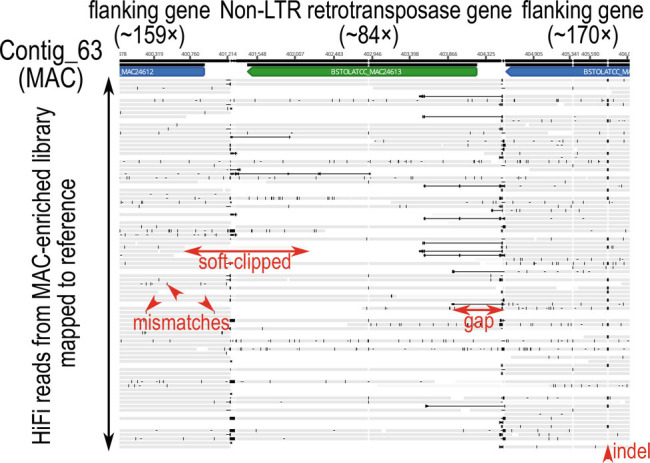
Non-LTR-retrotransposon sequences in both the germline and somatic genomes. Window of mapped HiFi reads from sucrose gradient-purified MACs (gray) spanning a retrotransposon gene with both an AP endonuclease domain and a reverse transcriptase domain (from rnd-4_family-193). Only sequence columns with <90% gaps are shown.

Three retrotransposon repeat families in the MAC + IES assembly were classified by RepeatClassifier, namely “LINE” or “LINE/RTE-X” (*SI Appendix*, Table S5). Two of these were more closely related, with numerous very high identity sequences (>97%) (*SI Appendix*, Fig. S7*A*), suggesting recent radiation of two related retrotransposon elements, while the third was more divergent (*SI Appendix*, Fig. S7*B* and *SI Results “Diversity of MAC-limited non-LTR retrotransposon-derived repeats”*). All of *Blepharisma*’s MAC genome-encoded transposases appear domesticated (i.e., have no TIRs) (37), and none have replicated to the same extent as the retrotransposon-derived repeats in this genome. Unlike the Bogo and BstTc1-derived elements, more retrotransposon-derived sequences, containing the reverse transcriptase domain RVT_1, were detected in the *Blepharisma* MAC genome than in assembled IESs (Fig. 3*E* and *SI Appendix*, Table S5). However, genes in IESs may be undercounted because of lower completeness of the germline vs. somatic assembly; indels caused by the lower accuracy of the uncorrected long reads used to assemble IESs that prevent prediction and shorter total length of IESs than somatic sequence. Consistent with them being true somatic sequences, mappings of error-corrected long reads from a MAC-enrichment library spanned well into flanking regions (Fig. 4 and *SI Appendix*, Fig. S8 *A* and *B*). In each repeat family, some loci showed sharp dips in coverage compared to flanking regions, suggesting that the elements are partly excised as IESs (Fig. 4), while other loci did not (*SI Appendix*, Fig. S8*B*). Despite this partial excision, coverage of such sequences is well above residual IES retention for MAC-enriched DNA (retention scores ≤0.02, *SI Appendix*, Fig. S1*B*). Several retrotransposase coding sequences themselves contained IESs (*SI Appendix*, *SI Results* “Parts of endonuclease domains in retrotransposase genes are excised as IESs”, *SI Appendix*, Fig. S7*C*).

Twenty-nine genes in the main somatic assembly encoded full or partial copies of reverse transcriptase domain RVT_1 (37). The four longest retrotransposon genes also encoded an N-terminal apurinic/apyrimidinic endonuclease (Exo_endo_phos_2) domain upstream of RVT_1. This domain pair is characteristic of some proteins from non-LTR retrotransposons/LINE-like transposable elements, e.g., the BS element from *Drosophila melanogaster* (UniProt Q95SX7) (43, 44). In contrast to the development-specific upregulation of retrotransposon genes in *Tetrahymena* (45) and *Oxytricha* (30), expression of *Blepharisma* genes encoding proteins containing RVT_1 or Exo_endo_phos_2 domains was negligible in starved cells and throughout a post-conjugation developmental time series, for both germline-limited and somatic copies (*SI Appendix*, Fig. S6) (37). The only exception was a somatic APEX1 protein homolog (BSTOLATCC_MAC3189). APEX1 is involved in DNA repair (46), and Blastp best matches of this *Blepharisma* protein to GenBank’s NR database are other similarly annotated proteins.

### Development-Specific 24 nt sRNAs Are Likely scnRNAs in *Blepharisma stoltei*.

Development-specific sRNAs play a role in marking sequences for excision or retention in other ciliates. To identify such sRNAs in *Blepharisma*, two complementary mating types of *Blepharisma* (strains ATCC 30299 and HT-IV) were separately gamone-treated and mixed to initiate conjugation, then sampled for sRNA-seq, mRNA-seq, and morphology over a 38 h time course. Expression patterns of somatic genes from mRNA-seq and the morphological staging have been reported in our sister report on the MAC genome (37). Briefly: after mating types were mixed (0 h), cells paired, produced gametic nuclei by meiosis, and exchanged them (2 to 18 h), followed by karyogamy (18 to 22 h) and development of the zygotic nuclei to new MACs (22 h onward). At 38 h, about a third of observed cells were exconjugants.

**Fig. 5.**
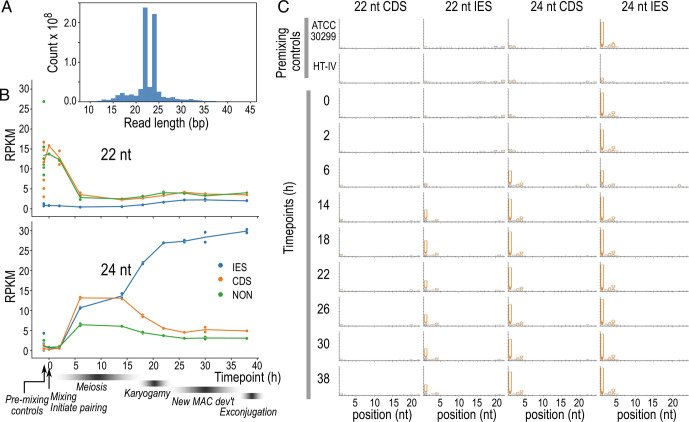
Development-specific, 24 nt sRNAs are likely scnRNAs in *Blepharisma*. (*A*) Read length histogram for all sRNAs in the time series. (*B*) Relative expression (RPKM units, vertical axis) of 22 and 24 nt sRNAs mapping to different feature types across time series: blue—IES, orange—CDS, green—all other regions not annotated as IES or CDS (including UTRs and intergenic regions which are difficult to delimit exactly with available data). Timing of developmental stages inferred from morphology are labeled below (ref. 37). (*C*) Sequence logos for 22 and 24 sRNAs mapping to CDS and IES features in controls and different time points (rows). See also *SI Appendix*, Fig. S9.

The most abundant sRNA length classes were 22 and 24 nt, comprising 32% and 30% of the total reads, respectively (Fig. 5*A*). This is consistent with other model ciliates, where Dicer-generated, mRNA-derived siRNAs employed in gene silencing are typically 21 or 22 nt long, whereas development-specific sRNAs are distinct and consistently ≥2 bp longer (19, 47).

Developmental dynamics of the 24 nt *Blepharisma* sRNAs resembled scnRNAs of other species. Coverage of 24 nt sRNAs mapping to all feature types initially increased from 2 to 6 h and plateaued until 14 h. Coverage over IESs increased further from 14 h to 22 h, reaching ~25 RPKM by the last time point (38 h), whereas coverage declined over coding sequences (CDSs) and other genomic regions (“NON”) after 14 h. The initial increase across all feature types coincided with meiotic stages iv to viii of ref. 35 (37), whereas the divergence between IESs and the rest of the genome corresponded to the onset of karyogamy (Fig. 5*B*). Furthermore, the relative coverage of 24 nt sRNAs was lower over periodic IESs and BogoMITE IESs compared to other types of IESs (*SI Appendix*, *SI Results “Putative scnRNAs have lower coverage over periodic IESs and BogoMITE IESs”*). In contrast, 22 nt sRNAs were initially abundant (albeit with high variance) at CDS and NON regions but low (<1 RPKM) at IESs and declined sharply to <5 RPKM in all features from 6 h onward (Fig. 5*B*).

*Blepharisma* 24 nt sRNAs had a strongly conserved 5′-U base preference, like scnRNAs in other ciliates (21, 47, 48). For 24 nt sRNAs mapping to IESs, all time points showed conserved 5′-U except for a slight decrease at 6 h (Fig. 5*C* and *SI Appendix*, Fig. S9*B*). 24 nt sRNAs mapping to CDSs only showed 5′-U bias after 6 h. We interpret this to mean that 24 nt sRNAs mapping to IESs were predominantly scnRNAs at all time points, whereas those mapping to CDSs initially comprised siRNAs and other types of sRNAs, before being dominated by scnRNAs from 6 h onward. In contrast, 22 nt sRNAs mapping to CDSs showed no base biases at any time point, whereas 22 nt reads mapping to IESs had a moderate 5′-U bias only from 6 h onward. The latter may represent true 22 nt scnRNAs or fragments of originally 24 nt scnRNAs.

## Discussion

*Blepharisma stoltei* belongs to the earliest-diverging lineage of ciliates sequenced to date. In most respects, genome editing in *Blepharisma* is more similar to oligohymenophoreans than spirotrichs, suggesting that characteristics shared with the former, such as TA-bound IESs, a PiggyBac excisase, and scnRNAs targeting IESs for excision, were likely to have been present in the ciliate common ancestor. Nonetheless, some characters may be disjunct with phylogeny, in particular the periodic length distribution of short IESs, which is shared only with the genus *Paramecium*. *Blepharisma* also provides fresh observations, notably the recent proliferation of nonautonomous MITEs that have autonomous counterparts in the same genome and of retroelements in the somatic genome. The former illustrate how MITEs could be an intermediate stage in the origin and proliferation of IESs.

### Comparison to IESs in Other Ciliates.

Most *Blepharisma* IESs are short, TA-bound, and intragenic, more similar to *Paramecium* than *Tetrahymena* or spirotrichs. The most striking parallel is the sharply periodic length distribution of short IESs, with peaks every ~10 bp, coinciding with the DNA helical turn. This implies that the *Blepharisma* excisase complex has similar geometric constraints as those proposed for *Paramecium* (2) (*SI Appendix*, SI Results “Periodic IES length distribution”). Compared to *Paramecium*, *Blepharisma* “periodic” IESs are longer on average and do not have a “forbidden” second peak, but the last peak (~110 bp; Fig. 1*C*) is still below the upper limit where such periodicity would be expected given the properties of DNA (Figure 7 of ref. 2). In contrast, *Tetrahymena thermophila* IESs have a continuous distribution (average length ~3 kbp) (1, 39), while *Oxytricha trifallax* nonscrambled IESs (length ~20 to 100 bp) have weak periodicity (30). Periodicity is consistent with a single primary IES excisase, rather than multiple excisase families, which would smooth the length distribution. Along with the physical properties of the DNA double helix itself, chromatin accessibility and species-specific characteristics such as nucleosomal linker length may also contribute to the IES length distribution. It was recently reported that chromatin remodeling in *Paramecium* requires an ISWI homolog for correct IES excision; knockdown of this gene leads to less pronounced periodicity and excision of sequences whose length fall in the “forbidden” peak (49).

Longer, nonperiodic IESs of *Blepharisma* contain more repeats, including whole transposons, than short IESs. Unlike *Tetrahymena*, where 41.7% of high-confidence IESs comprise putative autonomous transposons (1), some of which can be grouped into families (45, 50), only a small fraction of *Blepharisma*’s long IESs encode transposases, and their length distribution is not unimodal but long-tailed, with distinct peaks representing individual abundant families (Fig. 2). In *Paramecium*, longer, repeat-containing MIC-limited DNA sequences are alternatively associated with imprecise fragmentation into shorter MAC chromosomes and telomeric capping, or imprecise DNA elimination and rejoining (11). For this reason, they have been treated as distinct from the short, periodic *Paramecium* IESs. Since chromosome fragmentation is more extensive in *Blepharisma*, with no apparent association other than DNA composition, we refer to any MIC-limited sequence flanked on both sides by MAC-destined sequences as an IES.

Germline-specific repeats and transposons across *Paramecium* spp. have recently been surveyed (12) but were likely underestimated because such repeats are difficult to assemble from short-read data even with high coverage, as we saw with *Blepharisma* BogoMITE elements (*SI Appendix*, *SI Results* “IES assembly from short vs. long reads”, *SI Appendix*, Fig. S1*A*). The use of long read sequencing in this study hence helped to improve the detection of autonomous transposons in *Blepharisma* vs. *Paramecium*, but there is room for improving our MAC-scaffolded assembly method. Our method is less effective at assembling IESs that are longer than the average read length (39). Increased read lengths and better assembly algorithms should improve this. In future it will be desirable to develop functionality to assemble, annotate, and analyze other eliminated sequences at chromosome boundaries, as well as possible MIC-limited chromosomes.

The dynamics of *Blepharisma* 24 nt sRNAs are consistent with the scnRNA turnover model, where RNA intermediates are produced from both IESs and MDSs (51, 52), but those from MDSs are selectively degraded, allowing the remaining scnRNAs to mark IESs for excision. *Blepharisma* 24 nt sRNAs mapping to IESs increase more than those mapping to CDSs during post-conjugation development (Fig. 5*B*), complementing our finding that homologs of scnRNA biogenesis proteins, Dicer-like (Dcl) and Piwi proteins, are highly upregulated during development (37). Furthermore, there is higher coverage of *Blepharisma* scnRNAs in longer (presumably younger) IESs than in short (~older) periodic IESs, mirroring the situation in *Paramecium* where there is little to no dependence on scnRNAs for excision of shorter, older IESs but considerable dependence for longer, younger IESs (12, 53).

The longer an IES, the more likely it will contain a promoter by chance or contain one from a transposase gene, thus giving rise to such sRNAs. This would explain the low 24 nt sRNA levels from BogoMITE IESs compared to their autonomous counterparts (*SI Appendix*, Fig. S9*A*), though removal of both is essential. In contrast to the abundant 24 nt sRNAs from Bogo transposons, expression of these and other transposase genes in mRNA-seq is negligible (*SI Appendix*, Fig. S6). This raises the possibility that active, transcribed *Blepharisma* transposons are in fact silenced, turning most of their transcripts into 24 nt sRNAs. This is an alternative to the conventional role of scnRNAs in targeting DNA for excision, but congruent with the role of sRNAs in transposon silencing in other eukaryotes, from which the scnRNA biosynthesis enzymes originated (20).

### Are MITEs a Missing Link in the IBAF Model?.

The prevailing Invasion-Bloom-Abdication-Fade (IBAF) model for the evolution of IESs hypothesizes that they originate from cut-and-paste DNA transposons that invade and proliferate (“bloom”) in the germline genome (4). Transposon proliferation stops (“abdication”) when its transposase is domesticated by a host promoter, releasing the transposons from purifying selection, whereupon their sequences erode by drift (“fade”). Depictions of the IBAF model usually show all the transposons expressing transposases during “bloom”, i.e., functioning as autonomous transposons (4, 54). This is reasonable for *Tetrahymena* and *Oxytricha*, which have hundreds of germline-encoded transposases that vastly outnumber those in the somatic genome (*SI Appendix*, Table S4). However, *Blepharisma* and *Paramecium* only have a few dozen transposases, although germline-limited transposases may be underestimated, especially for short-read assemblies.

This discrepancy can be resolved by taking MITIESs (MITE IESs) into account. In *Blepharisma* this is best exemplified by the few autonomous Bogo transposon copies compared to thousands of nonautonomous BogoMITEs. The narrow length distribution of BogoMITEs, their high sequence identity, and occasional nested insertion inside unrelated IESs are the clearest illustrations to date of recent MITE proliferation. Bogo is also the first Pogo/Tigger transposon found in a ciliate germline genome; this subfamily is known to be especially prone to MITE formation (55, 56). The prevalence of IESs bound by TIRs, including numerous palindromic IESs (*SI Appendix*, Figs. S2*D* and S3), also suggest many more *Blepharisma* IESs are MITE derivatives.

In *Paramecium* spp., MITEs of the Thon and Merou transposons have been identified but only numbered about a dozen copies per genome, and their transposases belong to a different transposase family than Bogo (Fig. 3). The most abundant mobile IES family in *Paramecium*, FAM_2183, is probably a MITE but its autonomous counterpart was not reported (12). MITEs as transposon/IES life cycle intermediates can hence explain why *Blepharisma* and *Paramecium* have few MIC-encoded transposases compared to *Oxytricha* and *Tetrahymena* but nevertheless tens of thousands of IESs.

**Fig. 6.**
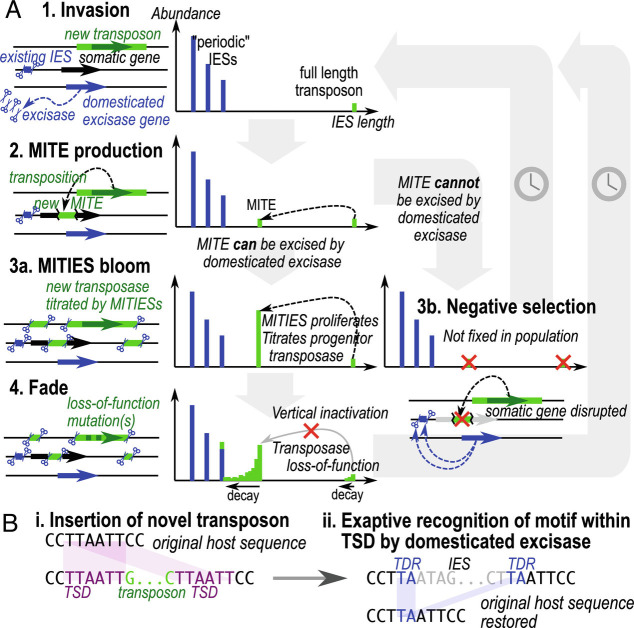
Model for IES evolution in a ciliate genome with an existing domesticated excisase. (*A*) Graphs depict IES length distribution. (*1*) Invasion of germline genome by full-length transposon (green); existing IESs (blue) are excised by domesticated excisase. (*2*) New transposon produces MITIES which are both MITES and IESs. (*3a*) If MITIES can be excised by domesticated excisase, they proliferate and titrate the progenitor transposase. (*4*) Proliferation of MITIES favors vertical inactivation of the full-length transposon; loss of function stops production of new MITIES, leading to eventual decay. (*3b*) If the MITE cannot be excised by domesticated excisase (i.e., it is not an IES), it is more likely to cause deleterious mutations upon insertion and is therefore selected against and does not reach fixation. (*B*) If a transposon TSD contains a submotif that can be recognized by the domesticated excisase, it can theoretically be excised cleanly without leaving a “footprint”, avoiding potential frameshift mutations. Although they are potentially involved, sRNAs, long noncoding RNAs, and chromatin state are not shown, for clarity and because the timing of their origin relative to the excisase is unknown.

MITEs also provide a mechanism for the self-limitation of transposon/IES proliferation (Fig. 6*A*). When MITEs outnumber the autonomous transposon, active transposase protein is more likely to bind to target sites in MITEs than the full-length transposon (“titration”), hindering the replication of the autonomous version, giving time for loss-of-function mutations to inactivate the transposases (“fade”). This “vertical inactivation” scenario (57) was already discussed in the original IBAF proposal (4), but no plausible examples from ciliates were then known.

### Why Does the *Blepharisma* Somatic Genome Contain Retrotransposon Sequences?.

Transposon-related sequences are typically germline-limited in other model ciliates, which was formerly interpreted as successful “genome defense” keeping them out of the somatic genome (1, 29, 30, 45, 58). Counter to this, we found several retrotransposon-derived sequences in the *Blepharisma* MAC genome (Fig. 4 and *SI Appendix*, Fig. S7 and Table S2). Some show signs of partial excision or possible absence of the locus in part of the population, but plenty have uniform coverage typical of somatic sequences.

Recent retrotransposon proliferation in the soma and patchy distribution of different somatic transposase classes across ciliates (*SI Appendix*, Table S4) (37) might suggest that “genome defense” is leaky. However, we favor a simpler hypothesis, described in the next part of the discussion, that evolution has determined which sequences are now observed in the somatic genome. We also conjecture that if foreign DNA lacks suitable target sites recognized by the excisase, it might still be marked by scnRNAs but fail to be excised or only be partially excised (e.g., the IESs in *SI Appendix*, Fig. S7*C*). Such DNA would still be deleterious if inserted intragenically.

Somatic MACs may generally be unable to repress mobile elements by heterochromatinization like germline MICs and other eukaryotic nuclei. In *Tetrahymena*, most MAC DNA is not associated with classical heterochromatin marks (23), while in *Paramecium* MACs, H3K27me3 is not associated with transcription repression, despite being a classic heterochromatin mark in multicellular eukaryotes (59). In such a permissive expression environment, selection against mobile elements that are not already excised as IESs may be especially effective, unless they are relatively transcriptionally inactive like the *Blepharisma* retroelements. On the other hand, regular *Blepharisma* stock culture passaging maintains a small effective population size, which would counteract selection against mobile element accumulation in the soma.

The genome defense model may lead one to dismiss IES retention in the somatic genome as excisase inefficiency or MIC contamination of the library; however, IES excision is not all-or-nothing but a continuum. Experimental evolution experiments in *Paramecium* suggest IES retention variability is itself a plastic and evolvable trait with consequences for somatic genotypic diversity (60, 61). Assembly algorithms tend to present an oversimplified, “pristine” view of somatic genomes, because they collapse repetitive and lower-coverage regions, which are characteristic of mobile elements and partially retained IESs. Accurate long read sequencing, haplotype-aware assemblers, and sequence graphs will all play a role in building a more realistic picture of somatic genome heterogeneity.

### Is “Genome Defense” a Flawed Analogy?.

The IBAF model also does not explain how ciliates can consistently and precisely excise novel mobile elements from different transposon families that invade the germline genome. The domesticated excisases of *Paramecium* (6), *Tetrahymena* (7), and *Blepharisma* (37) belong to the PiggyBac family. Except for *Tetrahymena* Tpb2, PiggyBacs are known to perform seamless excision, where the host sequence after transposon excision is identical to that before insertion (62). This would make them the ideal progenitor for IESs within coding sequences; indeed, PiggyBac transposons are also known to produce MITEs (63, 64). By extension, the first IESs probably originated from PiggyBac transposons. But what about subsequent invasions by other transposons that leave behind “scars” upon excision? Such imprecision would cause deleterious frameshift mutations in coding regions. How can they invade the germline genome and yet avoid deleterious effects?

Part of the answer lies in the “hijacking” model proposed from *Paramecium* (2, 12), whereby the domestication of PiggyBac transposase changed the dynamic for subsequent transposon invasions. The first domesticated PiggyBac would have been selectively advantageous for the ciliate, either by excising existing transposons or a shorter DNA sequence that happens to interrupt a critical gene or its expression. New transposons would persist as IESs only if they also encode a seamless excisase or if they can also be recognized and cut by the exapted PiggyBac enzyme. The latter favors the invasion of transposons that produce a TSD containing a submotif recognized as a cut site by PiggyBac (Fig. 6*B*). The similarity between IES and transposon boundaries would hence not be due to common origin or sequence evolution after IES fixation in the germline (4) but rather because of selection for transposons whose TSDs already match the excision site preferences of domesticated PiggyBac. Analogous exaptation of TSDs for excision has been demonstrated in another context: independent origin of introns from MITEs in at least two different eukaryotes, where one of the TSDs produced upon MITE insertion was coopted as an intron splice site (65). Cross-talk between different (albeit related) transposases for MITE transposition has also been documented (66).

We further argue that the term “genome defense” is teleological and confuses cause and effect, because “defense” implies that it acts in the interest of the host organism, whereas domesticated excisases actually facilitate mobile element accumulation in the germline by shielding them from selection by effective exclusion from the somatic genome. *Tetrahymena* is the exception that proves the rule: its domesticated excisase appears to be imprecise; correspondingly, most of its IESs are intergenic, because intragenic IESs have been efficiently removed by selection (3, 67). The origins of gene silencing by DNA methylation in vertebrates have also been reinterpreted with similar reasoning. Vertebrates have high levels of CpG methylation that inactivates transposons, which was thus proposed to “compensate for” transposon proliferation in eukaryotic genomes, similar to the argument of genome defense (68). When seen from a nonteleological perspective, however, it is precisely because CpG-mediated transposon inactivation is so effective at preventing exposure to selection that transposons persist in the genome (69).

Why do we credit developmental DNA elimination with defending the genome, when natural selection has been doing the hard work? Apart from technical biases during genome assembly, there is also sampling bias by using lab strains. These are often clonal and largely homozygous; if so, we would not observe accumulation of strongly deleterious foreign DNA that actually needs defending against, but only IESs that have reached fixation and that are already efficiently excised and nondeleterious. Purifying selection against deleterious IESs has had to be indirectly observed, e.g., in the lack of intragenic IESs in *Tetrahymena*, where excision is imprecise (1), and the statistical depletion of IES-like sequences in the *Paramecium* somatic genome (25). Similar evolutionary logic applies to prokaryotic CRISPR defense systems, where hidden fitness costs (autoimmunity) have been underestimated because those individuals are removed by selection (70), hence the phenomenon is easily misinterpreted as inheritance of acquired traits (71).

## Conclusion

Given the phylogenetic position of *Blepharisma*, several characteristics of its genome editing that are shared with oligohymenophorans (especially *Paramecium*) appear to represent the state of genome editing in the common ancestor of these ciliates: i) enrichment of TA motifs at IES boundaries; ii) a distinct, development-specific size class of sRNAs resembling scnRNAs with conserved 5′-U that may target DNA for elimination; iii) the presence of a domesticated PiggyBac excisase; iv) and possibly also the periodic length distribution of short IESs. Nonetheless, the diversity observed in ciliate species studied thus far suggests that many traits may be plastic over evolutionary time and not strongly phylogenetically constrained. There remain nine class-level taxa for which no draft MIC genome is available. Discovery of abundant MITEs alongside their autonomous counterpart was also serendipitous given that we expected MITIES to be a transient state, and allowed us to fill in important intermediates in the IES life cycle (Fig. 6). Most studies on ciliate developmental DNA elimination to date have focused on the underlying molecular mechanisms. In parallel, our view of the origins and evolution of this process should be expanded to include diverse representatives of the classes for which no draft germline genomes are available. In future, it will also be essential to investigate natural populations to better understand the evolutionary dynamics of genome editing.

## Materials and Methods

General reagents were analytical grade and purchased from Sigma-Aldrich or Merck unless otherwise indicated. See also *SI Appendix*, *SI Methods*.

### Ciliate Strain Origins and Cultivation.

The strains used were isolated from single cells and their original isolation localities were: *Blepharisma stoltei* ATCC 30299, Lake Federsee, Germany (72) and *Blepharisma stoltei* HT-IV, Aichi prefecture, Japan (73). Methods for cell cultivation and harvesting of material for sequencing are described in our sister report (37). The same report shows that the strain ATCC 30299 MAC genome is essentially homozygous. Previously, conjugation of the two strains resulted in normal cell maturation with healthy progeny and an immaturity period of about 13 to 17 cell divisions after conjugation (74). Strain ATCC 30299 has been grown for over 50 y and HT-IV for over a decade.

*Blepharisma stoltei* stocks (100 to 400 mL) have been maintained in several laboratories with regular subculturing every few weeks, feeding either bacteria or algae. In our laboratory in Germany, we have maintained stocks by subculturing 10 mL cells into 100 mL of fresh algae medium every 3 wk. To produce fresh algae medium, *Chlorogonium elongatum* grown in a TAP medium (75) was centrifuged (1,500 g; 3 min; room temperature), the spent medium was decanted, and the algal pellet was gently resuspended in SMB medium (76).

### Enrichment of MICs, Isolation and Sequencing of Genomic DNA.

*Blepharisma stoltei* ATCC 30299 cells were harvested and cleaned to yield 400 mL of cell suspension (1,600 cells/mL). This suspension was twice concentrated by centrifugation (100 g; 2 min; room temperature) in pear-shaped flasks and in 50-mL tubes to ~8 mL. Ten milliliters of chilled Qiagen Buffer C1 (from the Qiagen Genomic DNA Buffer Set, Qiagen no. 19060) and 30 mL chilled, autoclaved deionized water were added. The suspension was mixed by gently inverting the tube until no clumps of cells were visible and then centrifuged (1,300 g; 15 min; 4 ˚C). The pellet was washed with chilled 2 mL Buffer C1 and 6 mL water, mixed by pipetting gently with a wide-bore pipette tip, centrifuged (1,300 g; 15 min; 4 ˚C), and resuspended with chilled 2 mL Buffer C1 and 6 mL water by pipetting gently with a wide-bore pipette tip.

The nuclei suspension was layered over a discrete sucrose gradient of 20 mL 10% (w/v) sucrose in a TSC medium (0.1% (v/v) Triton X-100, 0.01% (w/v) spermidine trihydrochloride and 5 mM CaCl_2_) on top of 40% (w/v) sucrose in a TSC medium (77). Gradients were centrifuged (250 g; 10 min; 4 ˚C). Then, 10 to 12 mL fractions were collected by careful pipetting from above, and the nuclei were pelleted by centrifugation (3,000 g; 10 min; 4 ˚C). DNA was extracted from pelleted nuclei with the Qiagen Genomic tips 20/G and HMW DNA extraction buffer set (Qiagen no. 19060) according to the manufacturer’s instructions. DNA concentration was measured by the Qubit dsDNA High-Sensitivity assay kit. Fragment size distribution in each sample was assessed by a Femto Pulse analyzer.

DNA isolated from the MIC-enriched fraction on two separate occasions was used to prepare two sets of DNA sequencing libraries. A low-input PacBio SMRTbell library was prepared without shearing the DNA and was sequenced in the CLR (continuous long read) sequencing mode on a PacBio Sequel II instrument at the Max Planck Genome Centre Cologne, Germany (https://mpgc.mpipz.mpg.de/home/). Paired-end short-read libraries were prepared for four sucrose gradient fractions [top (T), middle (M), middle lower (ML), bottom (B)] and sequenced with 100 bp BGI-Seq paired-end reads on a BGI-Seq instrument.

### IES Prediction from PacBio Subreads.

PacBio subreads (CLR reads) from a *Blepharisma stoltei* ATCC 30299 MIC-enriched sample (ENA accession ERR6548140 (78)) were aligned to the somatic genome reference assembly (accession PRJEB40285) (37) with minimap2 v2.17-r941 (79), with options: -ax map-pb --secondary=no --MD. Mapped reads were sorted and indexed with samtools v1.10 (80) and then used for predicting IESs with BleTIES MILRAA v0.1.9, with options: --type subreads --junction_flank 5 --min_ies_length 15 --min_break_coverage 10 --subreads_pos_max_cluster_dist 5. The BleTIES pipeline has been previously described (39) and uses spoa v4.0.3 (81) for assembly. After inspecting the initial IES predictions (*SI Appendix*, Fig. S1*A*), we removed IES predictions with length <50 bp and retention score <0.075, which we judged to be more likely to be spurious or to have insufficient coverage for an accurate assembly.

TDRs at the boundary of a given IES were defined as a sequence of any length that was exactly repeated on both ends of the IES, such that one copy lies within the IES and the other in the MAC-destined sequence. Because the sequence is identical, it is not possible to determine from sequencing data alone where the physical excision of the IES would occur; such ambiguous excision junctions have been termed “floating IESs” (12). Therefore, TDRs were always reported starting from the left-most coordinate. If the TDR sequence contained 5′-TA-3′, the corresponding IES was also considered to be “TA-bound”, even if the TDR was longer than the 2 bp 5′-TA-3′ sequence. The expected:observed ratios for TDRs of different lengths were computed with empirical base frequencies (*SI Appendix*, *SI Methods “Probability of a pair of sequences”*).

Reconstructed IES sequences were computationally inserted into the MAC assembly with BleTIES Insert, to produce a hybrid MAC + IES assembly (78, 82), which approximates the part of the MIC genome that is collinear with the MAC.

### Identification and Comparison of IES Length Classes.

Visual inspection of the length distribution of BleTIES-predicted IESs showed sharp peaks every ~10 bp between ~65 and 115 bp. Peak calling on the graph of number of IESs (TA-bound only) vs. length (bp) was performed with the function find_peaks from the Python package scipy.signal v1.3.1 (83), with height cutoff 100. The ranges for each IES size class were defined with the width at half peak height. In *Paramecium tetraurelia*, where most IESs are TA-bound, the IES termini have a short, weakly conserved inverted repeat (2, 5). To search for similar motifs in *Blepharisma*, sequences flanking TA-bound IES junctions were extracted, with one from each pair reverse-complemented so that the sequences were always in the orientation 5′-(MDS segment)-TA-(IES segment)-3′. Sequence logos of the junctions (10 bp MDS, 14 bp within IES, not including the TA itself) were drawn for each IES length class with Weblogo (84). Only TA-bound IESs were used for the sequence logos because they could be aligned relative to the 5′-TA-3′ repeat, whereas for IESs bound by other types of junctions, there is no common reference point to align the boundaries of the IES.

### Identification of TIRs.

The BleTIES-assembled IES sequences for *Blepharisma* were used to identify exact, ungapped TIRs. Starting from the ends of the IES sequence immediately within the flanking TDRs, each base was compared to the reverse complement of the corresponding base on the opposite end for a match, extending the TIR until a mismatch was encountered, up to a maximum length of 25 bp. The expected:observed ratios for TIRs of different lengths were computed with empirical base frequencies (*SI Appendix*, *SI Methods* “Probability of a pair of sequences”).

### Developmental Time Series sRNA-seq.

Complementary *Blepharisma stoltei* mating strains ATCC 30299 and HT-IV were pretreated with Gamone 2 and Gamone 1 respectively, and then mixed to initiate conjugation as described previously; sRNA and mRNA were isolated from total RNA at the same time points [“Conjugation time course”, (37)]. sRNA libraries were prepared with the BGISeq-500 sRNA Library protocol, which selects 18 to 30 nt sRNAs by polyacrylamide gel electrophoresis, and sequenced on a BGISeq 500 instrument.

### sRNA Libraries Mapping and Comparison.

sRNA libraries (85) were mapped to the MAC + IES assembly with bowtie2 v2.4.2 (86) using default parameters. Total reads mapping to CDS vs. IES features were counted with featureCounts v2.0.1 (87). To account for different total sequence lengths represented by CDSs, IESs, and intergenic regions, the read counts were converted to relative expression values [reads per kbp transcript per million reads mapped, RPKM (88)] using the total lengths of each feature type in place of transcript length in the original definition of RPKM, with the following formula:

$$10^{9}\;\times \frac{\left(\text{reads mapped to feature type}\right)}{\left(\text{total reads mapped}\times \;\text{total length of feature type}\right)}.$$

Reads mapping to CDSs, IESs, or neither (but excluding tRNA and rRNA features) were extracted with samtools view, with 22 and 24 nt reads extracted to separate files. Read length distributions for each sequence length and feature type were summarized with samtools stats.

### Gene Prediction and Domain Annotation.

The tiny introns of *Blepharisma* are difficult to model with existing gene prediction software, hence RNA-seq data were mapped to the MAC genome to identify introns empirically, before using Intronarrator, a wrapper around AUGUSTUS (89) with similar parameters as for the *Blepharisma* MAC genome (*SI Appendix*, *SI Methods* “Gene prediction and domain annotation”). Domain annotations were generated with InterproScan 5.44-79.0. Sources for reference sequences for comparison of transposase-related domain content in ciliate MIC vs. MAC genomes are listed in *SI Appendix*, *SI Methods* “Gene prediction and domain annotation”).

### Repeat Annotation and Clustering.

Interspersed repeats were annotated in the combined MAC + IES assembly with RepeatModeler v2.0.1 (90), with manual curation of repeat families rnd-1_family-0 (corresponding to BogoMITE element) and rnd-1_family-73 (containing the BstTc1 transposon) (*SI Appendix*, *SI Methods* “Repeat annotation and clustering”) (91).

### Phylogenetic Analysis of Tc1/Mariner-Superfamily Transposases.

Representative CDSs for the Bogo and BstTc1 transposases were chosen from shortlisted copies of repeat families rnd-1_family-1 and rnd-1_family-73, respectively, on the basis of their length and predicted sequence domains (*SI Appendix*, *SI Methods* “Phylogenetic analysis of Tc1/Mariner-superfamily transposases”). These were aligned against an annotated alignment of the DDE/D domain (41) to identify the catalytic triad. For phylogenetic analysis of Tc1/Mariner-superfamily DDE/D domains, DDE_1 and DDE_3 domains from both MIC-limited and MAC genes were aligned against selected sequences from a published alignment (42), supplemented with additional *Paramecium* sequences from (29); the tree was inferred with FastTree v2.1.11 (92) using the WAG substitution model (*SI Appendix*, *SI Methods* “Phylogenetic analysis of Tc1/Mariner-superfamily transposases”) (93).

### Phylogenetic Analysis of Retrotransposon-Derived Sequences.

All the nucleotide sequences ≥500 bp for the repeat families identified by RepeatClassifier as LINE or LINE/RTE-x: rnd-1_family-273, rnd-1_family-276 and rnd-4_family-193 were aligned to one another with MAFFT v7.450 (automatic algorithm) (94), with the option to automatically determine sequence direction [via the MAFFT plugin for Geneious Prime (95)]. Since the alignment appeared to be poor between the rnd-4-family-193 sequences and the rest, we generated separate alignments for this family from the other two, also with MAFFT (E-INS-i mode). Maximum likelihood phylogenies were generated by PhyML (96) version 3.3.20180621 with the HKY85 substitution model.

## Supplementary Material

Appendix 01 (PDF)

## Data Availability

Genomic and additional related data have been deposited in ENA: Bioprojects PRJEB46944, PRJEB47200 (78, 85) and EDMOND doi: 10.17617/3.82, 10.17617/3.83 and 10.17617/3.JLWBFM (82, 91, 93) (See *SI Appendix* for details).
