# Supplementary material for: MITE infestation accommodated by genome editing in the germline genome of the ciliate Blepharisma
Source: Proc Natl Acad Sci U S A. 2023 Jan 20;120(4):e2213985120. doi: 10.1073/pnas.2213985120 (PMC9942856; doi:10.1073/pnas.2213985120)
Supplement: Supplementary file 1 — Appendix 01 (PDF) [file pnas.2213985120.sapp.pdf]

PNAS

Supporting Information for
MITE infestation accommodated by genome editing in the germline genome of the ciliate *Blepharisma*

Brandon Kwee Boon Seah, Minakshi Singh, Christiane Emmerich, Aditi Singh, Christian Woehle, Bruno Huettel, Adam Byerly, Naomi Stover, Mayumi Sugiura, Terue Harumoto, Estienne Carl Swart*

* Correspondence: Estienne Carl Swart
Email: estienne.swart@tuebingen.mpg.de

This PDF file includes:

Supporting text
Figures S1 to S9
Tables S1 to S6
SI References

Supporting Information Text

SI Results

IES assembly from short vs. long reads

IESs predicted from short read sequencing (ParTIES prediction) had higher retention scores than those from the long read library (BleTIES prediction) (Fig. S1A). However, IESs of size ~390 bp were largely absent from the ParTIES prediction, despite being an abundant size class in the BleTIES prediction (Fig. S1A). We attributed this to the repetitive sequence content in the ~390 bp IESs, which contained a highly conserved repeat element. ParTIES pools all putative IES-containing sequences in the whole library for reassembly before aligning the resulting contigs to the MAC to identify IESs (1), whereas BleTIES performs a separate targeted assembly for each IES. IESs which contain repetitive sequence content would be less likely to be accurately predicted by ParTIES because reads originating from different IESs would be assembled together into a hybrid contig that cannot be aligned to the MAC reference. Therefore, we used the BleTIES-predicted IESs for all subsequent analyses.

MIC sequence coverage and telomeric content

Per-IES retention scores from BleTIES had a median of 0.195, indicating that about 20% of the read library originated from the MIC genome (Fig. S1A). The average coverage of IES-containing reads underlying the predicted IESs was ~45x, but given the distribution of coverage values, we expect that more IESs could be assembled with greater sequencing coverage.

Reads originating from MIC (IES-containing) also contained less telomeric sequence (0.0228% of total read length) than MAC reads (IES-lacking) (2.98%). This was consistent with our previous observation that the MAC genome was fragmented into telomere-bound minichromosomes of ~130 kbp length, presumably, like other ciliates, from longer and more contiguous MIC precursor chromosomes (2).

Intragenic:intergenic ratio for different IES size classes

We also compared the ratios of intragenic to intergenic IESs for different IES size classes. We hypothesized that IESs belonging to the different ranges of IES size classes (periodic vs. non-periodic) may not have the same excision efficiency and would hence experience different selective regimes. For example, an intragenic IES with poor excision efficiency would be more negatively selected against than one with better efficiency. Compared to the overall intragenic:intergenic ratio of 2.33 (i.e., 70% intragenic), the IESs that belonged to the most abundant size class (~72 bp) were more likely to be intragenic (ratio 2.70, 73% intragenic, $p < 0.001$) than IESs as a whole (Table S6). Other size classes also had higher or lower ratios compared to the expectation but they were not statistically significant with our relatively conservative p-value cutoff.

"Cryptic" IESs in the MAC genome

In addition to conventional IESs, "cryptic" IES excision can occasionally occur, which is the low frequency excision of MDS sequences that are incorrectly recognized as an IES by the excision machinery (3, 4). Cryptic IESs were identified by mapping MAC reads back onto the MAC reference assembly and looking for pileups of deletions relative to the reference.

10,048 potential cryptic IESs ≥50 bp were detected, of which 5,635 (56.1%) were TA-bound, and 1,328 (13.2%) bound by other TDRs. The fraction of TA-bound cryptic IESs was lower than that of conventional IESs, which could be partially attributed to misprediction of cryptic IESs, because a lower coverage threshold was used to detect them compared to conventional IESs. IES-negative forms represent only a minority of reads at cryptic IES positions (median retention score

95%, i.e., only 5% of reads are IES-negative, Fig. S1D). Conversely, true IESs appear to be efficiently excised from MAC DNA (Fig. S1B). Nonetheless, the length threshold of cryptic IESs has a clear peak at ~72 bp, corresponding to the most abundant periodic IES size class found previously, and less prominent peaks at other size classes (82, 92, 101, 110). Furthermore, the fraction of cryptic IESs in the ~72 bp size class that were TA-bound was higher than average, at 66.0%. The sequence logo of TA-bound cryptic IES junctions did not show any obvious sequence bias apart from a T/A immediately after the “TA”, but the sequence logo for only the ~72 bp cryptic IESs shows a slight TTT bias from position 6 after the “TA” (Fig. S1F), which resembles the motif found in conventional IESs (Fig. 1D).

The most common TDR sequence of cryptic IESs was “TA”. Simple alternations of T/A were also common, as well as sequences containing “TTA” or “TAA”. Unlike the conventional IESs, cryptic IESs with “TAA” or “TTA” TDRs did not form a distinct size class at ~390 bp corresponding to the BogoMITEs, but were distributed similarly to the other cryptic IESs, with a peak at 72 bp (Fig. S1E). Therefore, it is likely that TTA/TAA could represent an intrinsic cut site preference of the domesticated excisase (or one of them).

Periodic IES length distribution

Paramecium and *Blepharisma* IESs differ in the following ways: (i) *Paramecium* IESs are shorter on average, with a first peak at ~27 bp compared to ~65 bp in *Blepharisma*; (ii) *Paramecium* has a “missing” second peak at ~36 bp; and (iii) the first peak (27 bp) is the highest in *Paramecium* and heights decrease thereafter (except for the “missing” peak), whereas in *Blepharisma* the second (72 bp) and sixth (110 bp) peaks are the highest. Geometric constraints of the excisase complex bound to DNA have been proposed as an explanation for the periodic length distribution and missing second peak of *Paramecium* (5). In this model, the shortest ~27 bp *Paramecium* IESs (peak 1) represent the length of DNA required to bridge the cleavage sites on two subunits of the excisase, whereas peaks 3 and above represent DNA with an intervening loop, and the ~10 bp length periodicity corresponds to the ~10 bp period of the dsDNA helix. Peak 2 is “forbidden” because it is too long for the active excisase complex but too short to form a loop. The periodic IES lengths in *Blepharisma* can also be explained by this model because the last periodic peak (110 bp) is still below the persistence length of DNA, however they are not as short as those in *Paramecium*. This suggests that all the periodic IESs *Blepharisma* IESs are also looped, but that their excisase is unable to operate on the very short loops that may occur in *Paramecium* (down to 44 bp).

The secondary maximum peak at 110 bp may represent a historical wave of IES proliferation: Assuming that new IESs usually start out longer than 115 bp, they gradually decay in length with time. Once they reach the periodic length range, they are “captured” in optimal excision peaks lengths, and will eventually accumulate at the shortest-length peak (72 bp in *Blepharisma*, 27 bp in *Paramecium*), which represent the most abundant size class. Therefore, the uppermost periodic peak (110 bp) may contain IESs that proliferated sufficiently long ago that the IESs have been degraded to ~110 bp length, but not long enough to filter down to the shorter length peaks. Alternatively, it could represent a secondary conformational optimum for the excisase complex, in addition to the primary optimum at 72 bp.

Terminal inverted repeats (TIRs) and palindromic IESs

Considering only TA-bound IESs, boundaries of “periodic” IESs had a weak consensus 5’-<u>TA</u>T rrn ttt t-3’ (weakly conserved bases in lowercase), whereas IES from “non-periodic” peaks had other signatures, e.g., 5’-<u>TA</u>T Agn nnT TT-3’ for both ~153 and ~174 bp IESs. Despite their heterogeneity, TIRs were more common and longer than expected by chance, even with a strict criterion of no gaps or mismatches (Fig. S2D-S2F). Sequence clustering of long (≥10 bp) TIRs showed distinct TIRs associated with specific IES lengths. Additionally, 376 completely palindromic IESs were identified, of which 153 (40.7%) fell within the same ~228 bp length peak, despite comprising several apparently unrelated palindrome sequences.

Of the 376 palindromic IESs (≥90% identity in self-alignment) identified, 153 (40.7%) fell within the ~228 bp IES length peak observed before (225-231 bp, Table S1), although some palindromic IESs were within the periodic IES length range (Fig. S3). However, when clustered at 90% sequence identity, palindromic IESs in the ~228 bp length range actually fell into several clusters, suggesting that this peak was composed of several families of palindromic IESs which happened to have a similar length, rather than a single family. This was confirmed by pairwise distances and visual inspection of the multiple sequence alignment of the cluster centroids (Fig. S3B, S3C). Some clusters had recognizable homology to each other, but many were over 40% divergent.

Catalytic triad in DDE/D-superfamily transposases

Intact catalytic triads were observed for each of the families in DDE/D superfamily represented in the MAC genome: one of eight PiggyBac domains, four of a total of nine DDE_1/DDE_3 domains, three of five DDE_Tnp_IS1595 domains and five of six instances of the MULE domains. The presence of the catalytic triad in the MIC instances of these domains was more varied. None of the PiggyBac domains had a complete catalytic triad, though the longest cORF contained an almost complete catalytic triad, where the second Aspartate residue appeared to be translocated by one amino acid. For the DDE1/DDE_3 domain-carrying MIC protein, only fifteen of the forty-seven lacked the complete triad. Three of five MIC-limited DDE_Tnp_IS1595 domains and nine of ten MIC-limited MULE domains lacked the catalytic triad.

Diversity of MAC-limited non-LTR retrotransposon-derived repeats

Repeat families rnd-1_family-276 and rnd-1_family-273 defined by RepeatModeler/ RepeatClassifier had partially overlapping membership, but largely correspond to two clusters of related sequences. Between clusters, there was 60 to 69% pairwise nucleotide identity (Fig. S7A), but within clusters, sequence identities were very high (>97%). For example, in addition to the seven high-identity, ~4.1 kbp long copies of repeat family rnd-1_family-276 (Main Text), seven shorter sequences with high identity to these (>97%) were found at other genomic locations (five in the low-quality “cruft” MAC+IES contigs). Three > 3 kb sequences present on different MAC genome contigs are > 98% identity at the nucleotide level with additional high identity copies present in the “cruft” genome portion.

An additional retrotransposon-derived repeat family, rnd-4_family-193 (Table S5; Fig. S7B) was more distantly related (28-35% nucleotide identity relative to long sequences from the other two families) and more divergent within the family itself. Among the rnd-4_family-193 sequences classified, only one copy was relatively long (4.6 kbp), and no sequences showed additional long, high-identity matches as was observed for rnd-1_family-273 and rnd-1_family-276. The next longest rnd-1_family-193 sequences were ~2.0 kbp, and thus too short to encode a complete retrotransposase with both endonuclease and reverse transcriptase domains.

Parts of endonuclease domains in retrotransposase genes are excised as IESs

Six retrotransposon-derived sequences from repeat family rnd-1_family-273 contained a central IES that encoded almost half the amino acids of an Exo_endo_phos_2 endonuclease conserved domain (Fig. S7C). Excision of the IES during development thus knocks out the endonuclease domain in the somatic version of the gene. Furthermore, the repeat units as a whole had >99% identity to each other over their ~4.1 kbp length, and were flanked by dissimilar sequences (Fig. S7C). The similar lengths of these IESs (173 to 182 bp), their homologous location relative to the coding sequence, and their high sequence identity (>96%) all point to a replication of an ancestral retrotransposon which coincidentally contained a sequence recognized and excised as an IES. In two of these cases, the endonuclease and reverse transcriptase domains can be linked into a single reading frame when the IES is present (Fig. S7C).

Expression of non-LTR-retrotransposon-derived sequences

In *Tetrahymena* cells, retrotransposon transcription was below the detection limits in vegetatively growing and starved cells, first observed when meiosis occurs, disappearing with time (6). In *Oxytricha* expression of LINE retroelements is prominent well after meiosis and negligible prior to this (7). In contrast, the expression of the *Blepharisma* RVT_1 genes was negligible in starved cells and throughout development (2). Such barely detectable expression throughout development was not observed for any of the previously proposed putative domesticated transposase families in the *Blepharisma* MAC genome (2). However, none of *Blepharisma*'s putative domesticated transposases are anywhere near as abundant as the retrotransposon repeats in the MAC genome, let alone show signs of substantial recent replication.

Rate of development post-conjugation

The timing of sRNA expression and turnover in *Blepharisma* appeared to be slower than in a similar experiment in *Tetrahymena*, hence the earlier timepoints of our *Blepharisma* experiment captured intermediate stages not observed in *Tetrahymena*. At 6 h and 14 h timepoints in *Blepharisma*, MDSs have comparable 24 nt sRNA coverage to IES regions, whereas by 3 h after mixing of complementary mating types in *Tetrahymena*, about 80% of scnRNAs mapped to IESs (8).

Putative scnRNAs have lower coverage over periodic IESs and BogoMITE IESs

Relative expression levels of putative scnRNAs differed between IES size classes. Based on the IES length distribution and repeat content, we divided IESs into five groups: (1) short "periodic" IESs (≤115 bp), (2) BogoMITEs, because that was the most abundant family, (3) IESs with full-length Bogo transposons, (4) IESs with full-length BstTc1 transposons, and (5) all other IESs ("non-periodic"). BogoMITEs and periodic IESs had lower scnRNA coverage (max ~5 and 10 RPKM respectively) compared with nonperiodic IESs (~30 RPKM). The former were comparable to or even lower than expression levels over non-IES features (Fig. S9A). Nonetheless, scnRNA coverage of BogoMITEs and periodic IESs showed an initial increase then plateau, without the subsequent decline seen in non-IES regions. Bogo-containing IESs had similar scnRNA coverage to other non-periodic IESs, but BstTc1-containing IESs had higher coverage (Fig. S9A).

Because of the repetitive sequence content in IESs and the short sRNA length, it is possible that the expression levels calculated could be affected by mis-mapping. We reason that such mismapping would not influence the results described above, because "periodic" IESs (group 1) had low repetitive content, whereas the transposon-containing IESs (groups 2, 3, 4) each represented a single repeat family so any mismappings would be contained within the same group and count towards the same RPKM value.

Sequence motifs in putative scnRNAs

In *Tetrahymena*, scnRNAs have a strong bias for 5'-U and also for base A in the third last base (-3A bias), which is typical of Dicer, which leaves a 2 nt 3'-overhang after cleaving dsRNA (9). In comparison, *Paramecium* scnRNAs do not have a pronounced -3A bias, because (1) the cleavage site bias is not necessarily symmetrical for both ends, e.g., Dcl2 has bias for 5'-U/AGA, (2) subsequent selection for 5'-U during loading further selects against Dcl2 products with -3A, and (3) there are multiple Dcl paralogs each with their own cleavage site biases (10), unlike *Tetrahymena* which has only one. Presumably the situation is similar in *Blepharisma*, which has three Dcl paralogs (Fig. S7A of (2)), and where there is a clear 5'-U bias in 24 nt sRNAs, but also a slight -3A bias at some time points, e.g., at 6 h for 24 nt sRNAs mapping to IESs (Fig. 5C, Fig. S9B).

SI Materials and Methods

IES prediction from BGIseq short reads

BGIseq reads (100 bp, paired end) from MIC-enriched sample "AT10" (ENA accession ERR6501836) were mapped to the MAC reference assembly with bowtie2 v2.4.2 on local mode within the ParTIES pipeline (1). We modified ParTIES (based on v1.02) to use SPAdes v3.15.2 (11) instead of Velvet (12) to assemble IES+ sequences (https://github.com/Swart-lab/ParTIES, branch "custom" commit f04ad7e2), since Velvet kept crashing on our data.

MIC read binning and telomere annotation

Internally error-corrected circular consensus sequence (CCS) reads were generated from the above CLR library with PacBio ccs v4.2.0 (https://github.com/PacificBiosciences/ccs), with 26.1% of ZMWs generating CCSs. CCS reads were mapped to the MAC reference assembly with minimap2 with the same parameters as CLR reads, except for option -ax asm20. The mapping and IES annotation were used to calculate per-read IES retention scores. Reads were binned with the MILCOR module of BleTIES into putative MAC (score <0.1) and putative MIC (score >0.9). Total telomere sequence length in the binned MAC and MIC reads were calculated with a Python regular expression search for the telomere repeat 5'-CCCTAACA-3' and its reverse complement.

Accommodation of IESs in annotation feature tables

IESs in the MAC+IES genome assembly submitted to ENA were annotated as "iDNA" features, which was the closest-fitting feature type supported by INSDC feature tables (https://www.insdc.org/files/feature_table.html), although IESs are included in the Sequence Ontology (http://www.sequenceontology.org/browser/current_release/term/SO:0000671).

We also note that IES features can potentially create confusion for certain applications when an IES is present within a CDS feature. This is because in GFF3 and INSDC feature tables, introns are usually defined implicitly when a CDS is split into multiple segments, rather than being separately annotated. Therefore, with a MAC+IES genome assembly and gene annotations from public sequence databases like ENA, one has to be careful to check whether the CDS is interrupted by introns or IESs, or both.

IES retention scores in MAC enrichment library

PacBio HiFi reads from a MAC enrichment library were mapped to the *B. stoltei* MAC reference assembly with minimap2, sorted and indexed with samtools. Retention scores for previously predicted IESs in the MAC were calculated with BleTIES MILRET with default parameters.

Annotation of cryptic IESs

PacBio HiFi reads representing *B. stoltei* ATCC 30299 MAC DNA (ENA ERR5873334, ERR5873783) were mapped onto the MAC reference (GCA_905310155) with minimap2, and sorted and indexed with samtools as described above. The mapping was processed with BleTIES MILRAA with options --type ccs --fuzzy_ies --min_ies_length 15 --min_break_coverage 2 --min_del_coverage 2. IES predictions that overlapped with telomere regions and "cruft" contigs were removed. Those IES predictions that represented deletions relative to the reference assembly were considered to represent possible cryptic IESs. TDRs and sequence logos of cryptic IES junctions were defined and drawn as described above for conventional IESs.

Intragenic:intergenic IES ratios for specific IES size classes

We tested whether specific IES length classes were more or less depleted within gene features, compared to all IESs as a whole (two-tailed test, null hypothesis: all IES length classes have equal probability of being intragenic). The intragenic vs. intergenic membership of IESs was held constant, but their assigned lengths were randomly permuted (without replacement) to obtain 1000 pseudoreplicates. For each of the 10 IES size classes defined in Table S1, the p-value was calculated as the fraction of pseudoreplicates where the simulated number of intragenic IESs was more than the actual observed value. The uncorrected p-value threshold (0.05) was adjusted to 0.005 after applying a Bonferroni correction for 10 tests, yielding <0.0025 and >0.9975 as the two-tailed p-value thresholds.

Clustering of terminal inverted repeats (TIRs) and identification of palindromic IESs

Long TIRs (≥10 bp) were clustered by sequence identity to look for IESs of potentially related origin, using the cluster_fast algorithm (13) implemented in Vsearch v2.13.6 (14) at 80% identity and the CD-HIT definition of sequence identity (-iddef 0). For each resulting cluster of similar TIRs, the cluster centroid was used as the representative sequence shown in Fig. S2. TDRs associated with each cluster's IESs were grouped by length, and for each TDR length a degenerate consensus was reported with the degenerate_consensus function of the Bio.motifs module in Biopython v1.74.

Palindromic IESs were defined as IESs that align to their own reverse complement with a sequence identity ≥90% (matching columns over sequence length); this definition was less strict and permitted inexact matches unlike the TIR search, to allow for sequence divergence and assembly errors. IES sequences were aligned with the PairwiseAligner function from Bio.Align in BioPython v1.74, using global mode and parameter match_score = 1.0, with all other scores set to zero.

Palindromic IESs were clustered with Vsearch cluster_fast as described above, except that one sequence (BSTOLATCC_IES35757) was manually removed after inspection of results because it appears to contain two different nested palindromic sequences. Cluster centroids were aligned pairwise as above and used to calculate a matrix of edit distances (matching columns / alignment length). The distance matrix was clustered with average linkage clustering to produce a sequence distance dendrogram with the functions average and dendrogram from scipy.cluster.hierarchy v1.3.1 (15).

Comparison of intragenic:intergenic IES ratios

Intragenic vs. intergenic IESs were defined by overlap of predicted IES annotations with "gene" feature annotations on the MAC reference (ENA accession GCA_905310155), using Bedtools v2.30.0 (16) and pybedtools v0.8.1 (17).

To test whether the underrepresentation of IESs within gene features was statistically significant, compared to the null hypothesis of IESs and gene feature locations being independently distributed, we assumed that the number of intragenic IESs would follow a binomial distribution with individual probability equal to the fraction of the genome that is covered by gene features. The p-value of the observed number of intragenic IESs would then be equal to the cumulative probability density up to and including the observed value.

Probability of a pair of repeated sequences

Under a null model where all bases in a sequence are independently and identically distributed, the probability P_n of having any possible sequence of length *n* bounding a given sequence feature (either a TDR or a TIR) is the sum of probabilities of all possible sequences (each of which notated as *k*) of length *n*, squared: $P_n = \sum_{k \in K} p_k^2$ which can be transformed to $P_n = (\sum_{b \in B} p_b^2)^n$,

where B is the alphabet of bases and p_b is the individual probability of each base. The number of possible sequences k of length n is simply $|K| = |B|^n$.

The probability of having a repeat of length at least 2 is equal to the probability of having a repeat of length 2, because all cases of repeat length > 2 implicitly have a repeat of length = 2. Therefore, the probability of having a repeat of length exactly n, i.e., match in bases 1 to n, and mismatch on base n+1 is $P_n \times Pr\ (mismatch) = P_n \times (1 - \sum_{b \in B} p_b^2)$. The expected number of TDRs/TIRs in *Blepharisma* were calculated by using the empirical base frequencies of the MAC+IES genome assembly for p_b, and multiplying this probability by the number of IESs.

mRNA-seq read mapping

Reads were mapped with a version of Hisat2 (18) where the static variable minIntronLen in hisat2.cpp in the source code is lowered to 9 from 20 (https://github.com/Swart-lab/hisat2/; commit hash 86527b9). Hisat2 was run with default parameters and parameters --min-intronlen 9 --max-intronlen 30. It should be noted that spliced-reads do not span introns that are interrupted by an IES due to the low maximum length, however such cases are not expected to occur often.

Gene prediction and domain annotation

To predict protein-coding genes in IESs, non-IES nucleotides in the MAC+IES assembly were first masked with ‘N’s. The Intronarrator pipeline (https://github.com/Swart-lab/Intronarrator), a wrapper around Augustus (19), was run with the same parameters as for the *B. stoltei* MAC genome, i.e., a cut-off of 0.2 for the fraction of spliced reads covering a potential intron, and ≥10 reads to call an intron (2). Without masking, gene predictions around IESs were poor, with genuine MDS-limited genes (with high RNA-seq coverage) frequently incorrectly extended into IES regions. The possibility of genes spanning IES boundaries was not catered for.

Domain annotations for diagrams were generated with the InterproScan 5.44-79.0 pipeline (20) incorporating HMMER (v3.3, Nov 2019, hmmscan) (21).

For comparison of transposase-related domain content in MAC vs. MIC, reference sequences were obtained from public databases for *Paramecium tetraurelia* (https://paramecium.i2bc.paris-saclay.fr/files/Paramecium/tetraurelia/51/annotations/ptetraurelia_mac_51_with_ies/), *Tetrahymena thermophila* (http://www.ciliate.org/system/downloads/3-upd-cds-fasta-2021.fasta), and *Oxytricha trifallax* (https://oxy.ciliate.org/common/downloads/oxy/Oxy2020_CDS.fasta, https://knot.math.usf.edu/mds_ies_db/data/gff/oxytri_mic_non_mds.gff). IES gene prediction in *Blepharisma* was hampered by intermittent polynucleotide tract length errors, due to the assembly of IESs from PacBio CLR reads. To mitigate this, a six-frame translation of the MIC-limited genome regions was performed using a custom script, then scanned against the Pfam-A database 32.0 (release 9) (22) with hmmscan (HMMER), with i-E-value cutoff ≤10^{-6}. Domains were annotated from the MAC genome with three different methods: using published coding sequences (“cds” in Table S4), six-frame translations (“6ft”), and six-frame translations split on stop codons (“6ft_split”).

Repeat annotation and clustering

To evaluate the repetitive sequence content in IESs, we applied a repeat prediction and annotation to the combined MAC+IES assembly, instead of clustering whole IESs by sequence similarity. This was so that: (i) repeats shared between the MDS and IES could be identified; (ii) Complex structures such as nested repeats could be detected; (iii) repeat families were predicted *de novo*, permitting discovery of novel elements; (iv) repeats did not have to be strictly identical to be grouped into a family.

Interspersed repeat element families were predicted from the MAC+IES genome assembly with RepeatModeler v2.0.1 (default settings, random number seed 12345) with the following

dependencies: rmblast v2.9.0+ (http://www.repeatmasker.org/RMBlast.html), TRF 4.09 (23), RECON (24), RepeatScout 1.0.6 (25), RepeatMasker v4.1.1 (http://www.repeatmasker.org/RMDownload.html). Repeat families were also classified in the pipeline by RepeatClassifier v2.0.1 through comparison against RepeatMasker's repeat protein database and the Dfam database. Consensus sequences of the predicted repeat families, produced by RepeatModeler, were then used to annotate repeats in the MAC+IES assembly with RepeatMasker, using rmblast as the search engine.

The consensus sequences for rnd-1_family-0 and rnd-1_family-73 were manually curated for downstream analyses. For rnd-1_family-0 (BogoMITE) the original consensus predicted by RepeatModeler for rnd-1_family-0 was 784 bp long, but this was a spurious inverted duplication of the basic ~390 bp unit; the duplication had been favored in the construction of the consensus because RepeatModeler attempts to find the longest possible match to represent each family. For family rnd-1_family-73 (containing BstTc1 transposon), the actual repeat unit was longer than the boundaries predicted by RepeatModeler. In most IESs that contain this repeat (19 of 22), it was flanked by and partially overlapping with short repeat elements from families rnd-4_family-1308 and rnd-1_family-117, which are spurious predictions. Repeat unit boundaries were manually defined by alignment of full-length repeats and their flanking regions.

Terminal inverted repeats of selected repeat element families were identified by aligning the consensus sequence from RepeatModeler, and/or selected full-length elements, with their respective reverse complements using MAFFT (26) (plugin version distributed with Geneious).

TIRs from the Dfam DNA transposon termini signatures database (v1.1, https://www.dfam.org/releases/dna_termini_1.1/dna_termini_1.1.hmm.gz) (27) were searched with hmmsearch (HMMer v3.2.1) against the IES sequences, to identify matches to TIR signatures of major transposon subfamilies.

Sequence logos for Bogo and BogoMITE repeat boundaries

To generate sequence logos for the Bogo and BogoMITE repeat boundaries, full length sequences for repeat families rnd-1_family-1 (>1800 bp) and rnd-1_family-0 (between 385-395 bp) and their flanking 10 bp were extracted and reverse complemented if necessary to be in the same orientation. Both repeats have a poly-C run in the TIR whose variable length results in misalignment at the repeat boundaries. Therefore, to ensure that the TSDs are properly aligned, the TSD and first three bases of the TIR on each boundary flanking the repeats were identified with a regular expression ..ACTC or its reverse complement GAGT..; the sequences within those boundaries were aligned with the E-INS-i algorithm from MAFFT v7.475. Alignment columns comprising >90% gaps were removed. The degapped alignment was concatenated with the flanking TSD+TIR removed earlier, and then used to generate sequence logos of the repeat boundary regions with Weblogo v3.7.5.

Phylogenetic analysis of Tc1/Mariner-superfamily transposases

Repeat family rnd-1_family-1 was initially classified as a "TcMar/Tc2" family transposable element by RepeatClassifier. 30 full length copies (>95% of the consensus length) were annotated by RepeatMasker, all of which fell within IESs and contained CDS predictions. However, CDSs were of varying lengths because of frameshifts caused by indels, which may be biological or due to assembly error; nonetheless, the nucleotide sequences had high pairwise identity (about 98%, except for one outlier). We chose BSTOLATCC_MIC4025 as the representative CDS sequence for phylogenetic analysis because it was one of the longest predicted and both predicted Pfam domains (HTH_Tnp_Tc5 and DDE_1) appeared to be intact.

For repeat family rnd-1_family-73, the initial classification was "DNA/TcMar-Tc1". As described above, CDS predictions were of variable lengths, and the longest CDSs were not necessarily the best versions of the sequence because of potential frameshift errors. For phylogenetic analysis,

we chose BSTOLATCC_MIC48344 as the representative copy, because a complete DDE_3 Pfam domain was predicted by HMMER that could align with other DDE/D domains from reference alignments described below.

The representative CDSs of the rnd-1_family-1 and rnd-1_family-73 transposases were aligned with MAFFT (E-INS-i mode) against a published DDE/D domain reference alignment (Supporting Information Dataset_S01 of (28)) to identify the residues at the conserved catalytic triad and the amino acid distance between the conserved residues.

For the phylogenetic analysis of the DDE/D domains in the Tc1/Mariner superfamily, both MAC- and MIC-limited genes containing DDE_1 and DDE_3 domains were separately aligned for each Pfam domain with MAFFT v7.450 (algorithm: E-INS-i, scoring matrix: BLOSUM62, Gap open penalty: 1.53) and trimmed to the DDE/D domain with Geneious and incomplete domains were removed. As reference, 204 sequences from a published alignment (Additional File 4 of (29)) were selected to represent the 53 groups defined in that study, choosing only complete domains (with all three conserved catalytic residues) and all *Oxytricha trifallax* TBE and *Euplotes crassus* Tec transposase sequences. Thirteen *Paramecium* Tc1/Mariner DDE/D domain consensus sequences were added (Additional File 4 of (30)). Sequences were aligned with MAFFT (E-INS-i mode) and trimmed to only the DDE/D domain boundaries with Geneious. Phylogeny was inferred with FastTree2 v2.1.11 (31) using the WAG substitution model. The tree was visualized with Dendroscope v3.5.10 (32), rooted with bacterial IS630 sequences as the outgroup.

Data Availability

The annotated draft MAC+IES genome for *Blepharisma stoltei* strain ATCC 30299 is available from European Nucleotide Archive (ENA) Bioproject PRJEB46944 under accession GCA_914767885. IES sequences and annotations, MAC gene predictions with intervening IESs, and gene predictions within IESs are available from EDMOND, doi:10.17617/3.83 and https://bleph.ciliate.org. ENA accessions for sequencing data for the MIC-enriched nuclear fractions are: ERR6510520 and ERR6548140 (PacBio CLR reads); ERR6474675, ERR6496962, ERR6497067, ERR6501836 (BGI-seq reads). Small RNA libraries from developmental time series are available from ENA Bioproject PRJEB47200 under accessions ERR6565537-ERR6565561. Repeat family predictions and annotations by RepeatModeler and RepeatMasker are available from EDMOND, doi:10.17617/3.82. Alignment and phylogeny of Tc1/Mariner superfamily transposase domains are available from EDMOND, doi:10.17617/3.JLWBFM.

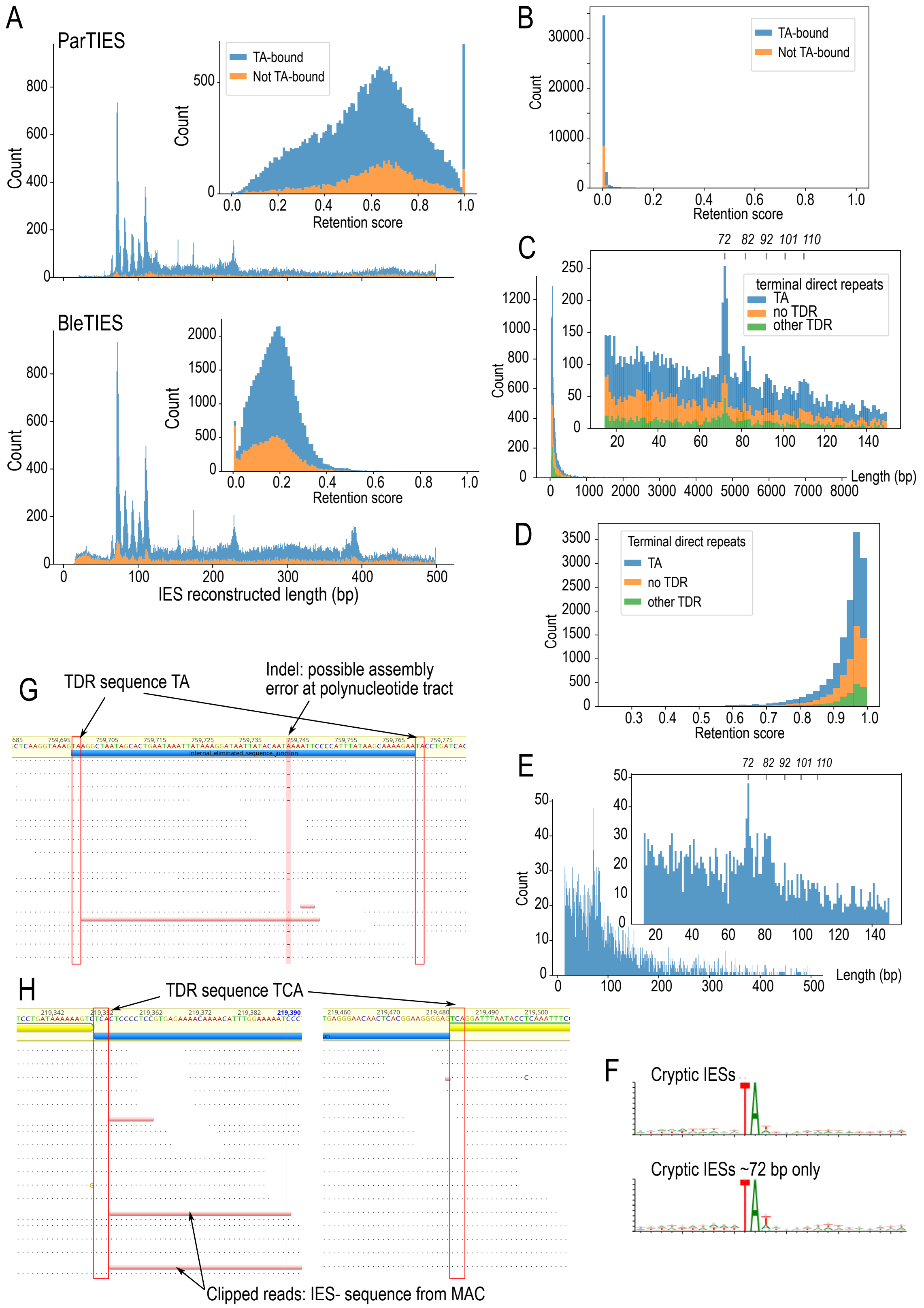

Fig. S1. Length distributions and retention scores for different IES assembly methods, MAC library, and cryptic IESs. (*A*) Comparison of IES reconstructions from MIC-enrichment library

sequenced with short reads by ParTIES (above) vs. from long reads by BleTIES (below). Main panels: IES length histograms up to 500 bp, insets: IES retention scores colored by TDR sequence type. Length peak at ~390 bp representing BogoMITE element is present in BleTIES reconstruction but not ParTIES. (*B*) Conventional IESs: retention scores computed from MAC-enriched library, sequenced with PacBio HiFi reads. (*C*) “Cryptic” IESs from MAC read library: length histogram, colored by TDR sequence type. (*D*) Retention scores of “cryptic IESs”. (*E*) Length distribution of “cryptic” IESs that contain “TTA” or “TAA” in their TDR, detail <500 bp, inset detail <150 bp. (*F*) Sequence logos of TA-bound “cryptic” IES junctions centered on the TA motif, for all cryptic IESs (above) and the subset in the ~72 bp size class (below). (*G*) Mapping pileup at IES with TA-containing TDR. For aligned reads in panels E and F, dots: bases identical to reference, dashes: gaps relative to reference, red bar: read clipping. (H) Mapping pileup at IES with non-TA-containing TDR.

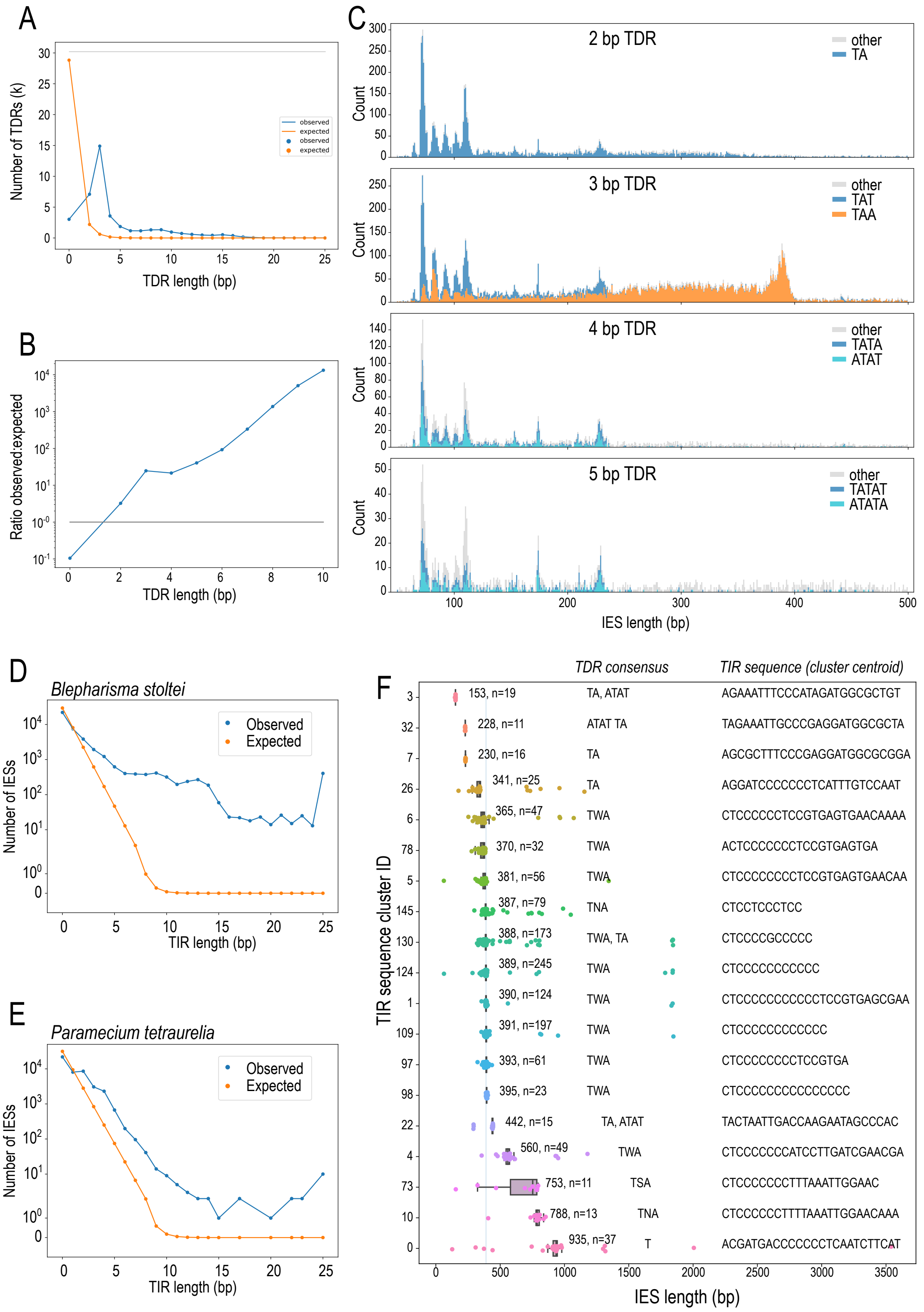

Fig. S2. IESs are bounded by heterogeneous direct and inverted terminal repeats. (*A*) Numbers of terminal direct repeats (TDRs) per TDR length observed (blue) vs. number expected by random chance if bases were independently distributed (orange). (*B*) Ratio of observed to

expected numbers of TDRs by length. (*C*) Length distributions of IESs containing TDRs of lengths 2, 3, 4, and 5 bp; the most abundant TDR sequences per TDR length are shown in color (sequences and their reverse complements are counted together, because TDRs could be encountered in either orientation, e.g., TAA/TTA), simple T/A alternations are in shades of blue. NB: plots in panel C have different vertical axis scales. (*D*) Observed IESs per terminal inverted repeat (TIR) length vs. expected number by chance alone. (*E*) Same as panel D but for *P. tetraurelia*. (*F*) Lengths (scatter-overlaid boxplot) of IESs containing long TIRs (≥10 bp), grouped by their TIR sequence (rows). Each TIR-cluster is annotated with the median IES length (bp), cluster size (n), TDR consensus sequence, and TIR representative sequence.

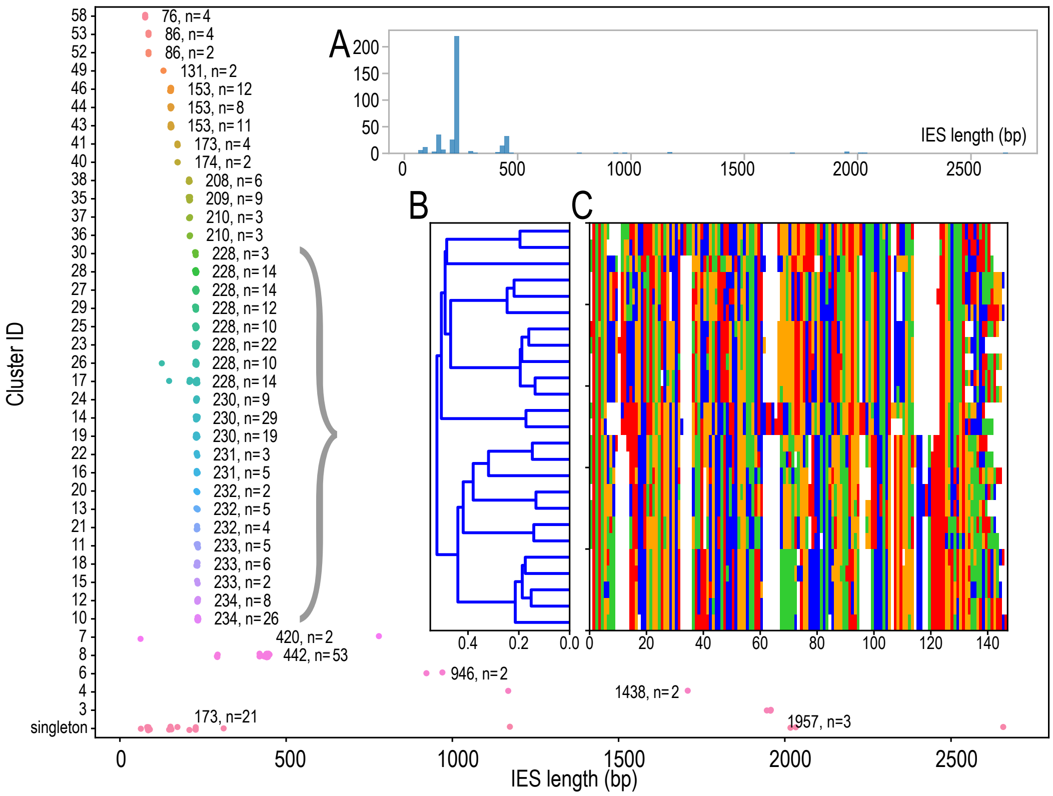

Fig. S3. Palindromic IESs clustering and length distribution. Strip plots of IES lengths for palindromic IESs (≥90% self-alignment identity), after they have been clustered by sequence identity (rows represent clusters). Each cluster is annotated with the median IES length and the cluster size. Insets: (*A*) Overall sequence length distribution histogram for all palindromic IESs. The most common length of palindromic IESs is ~230 bp. (*B, C*) Dendrogram of sequence distance and multiple sequence alignment of palindromic IESs with ~230 bp length to illustrate that they comprise several distinct clusters of sequences.

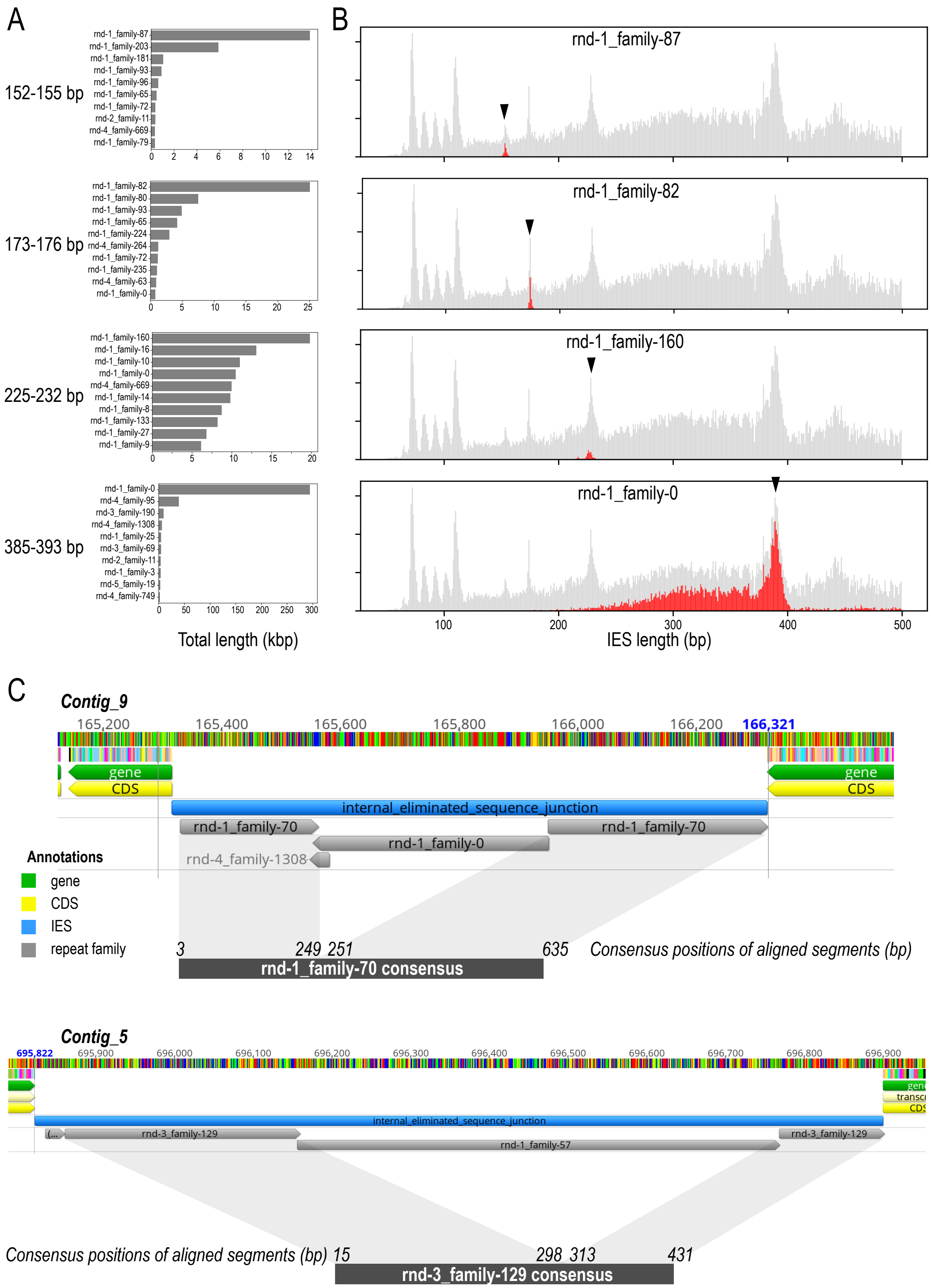

Fig. S4. Most abundant repeat families in non-periodic IES size classes. (*A*) Total lengths (horizontal axis) of the top ten repeat families per IES size class (panel rows). (*B*) Top repeat family (by sequence length) for each IES size class (panel rows); the total length covered by that

repeat family within IESs vs. the lengths of those IESs is shown in red, superimposed on the total sequence vs. IES length distribution of IESs in general (grey). Arrowheads mark centers of the size classes. (*C*) Examples of nested repeats within IESs. Nested elements can be recognized when the two outer repeat elements belong to the same family and align to consecutive parts of its family's consensus sequence, implying that the inner element has likely been inserted into the middle of an existing element. Coordinates of the split segments are relative to the repeat family consensus.

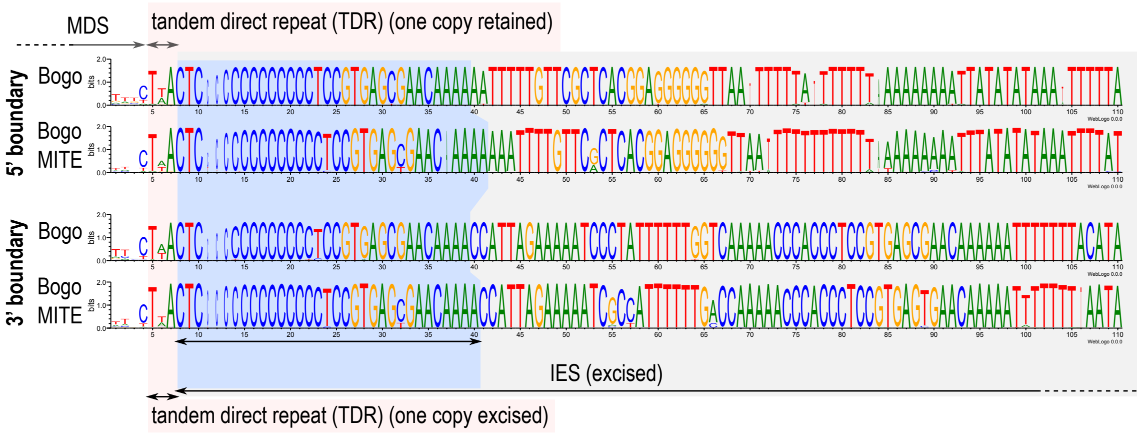

Fig. S5. Sequence logos for Bogo and BogoMITE repeat boundaries. Logos are aligned on the terminal inverted repeats (TIRs) and terminal direct repeats (TDRs). 3'-boundaries have been reverse complemented to show the TIRs. Sequence logos were generated from alignments of full-length, intact Bogo elements (>1.8 kbp) and BogoMITEs (between 385-395 bp), with columns comprising >90% gaps removed.

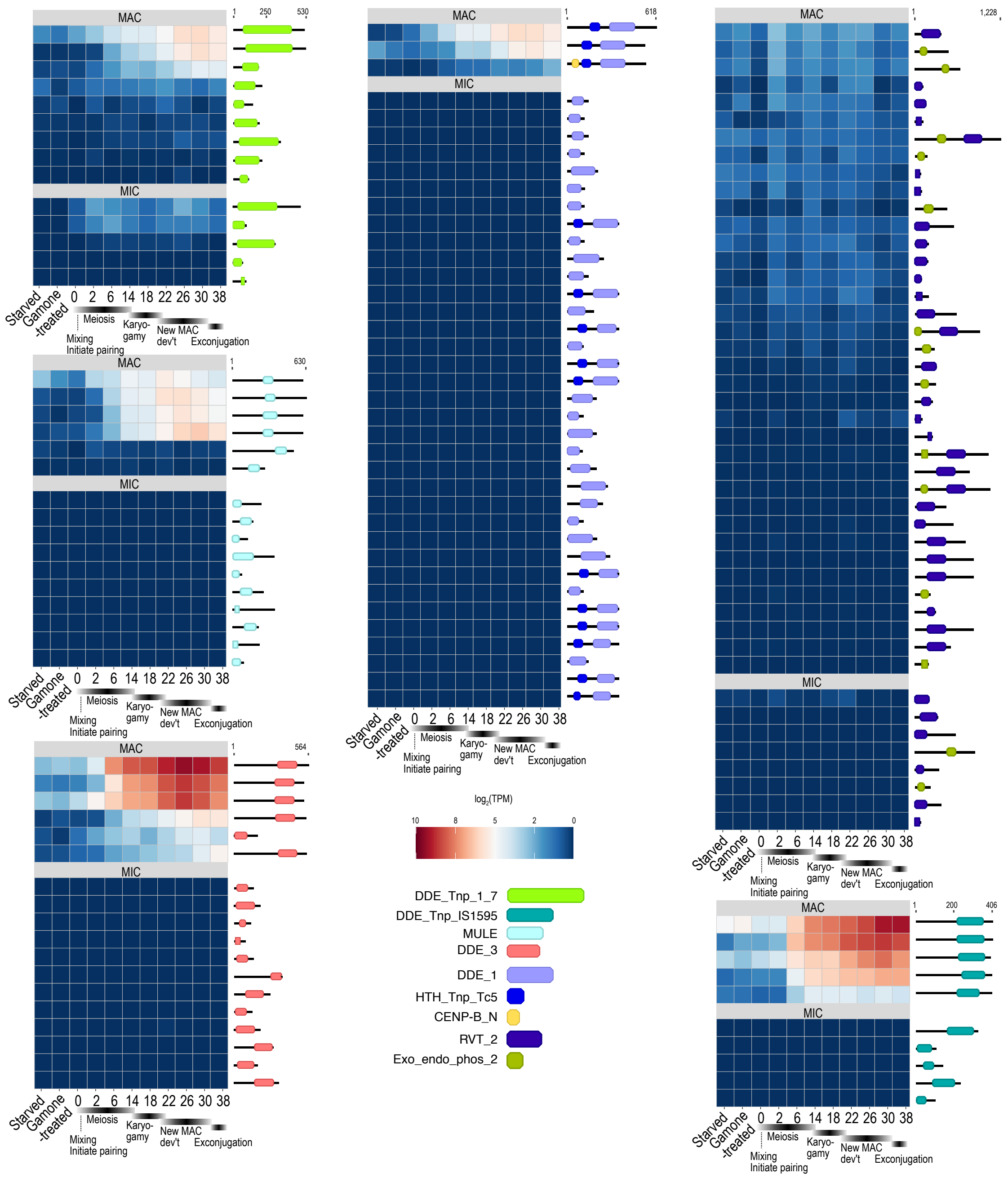

Fig. S6. Expression of genes with transposase domains. Comparison of expression levels for MAC- vs. MIC-limited transposase-related domains across developmental time series; heatmap color scaled to log(transcripts per million). Domain architecture shown diagrammatically.

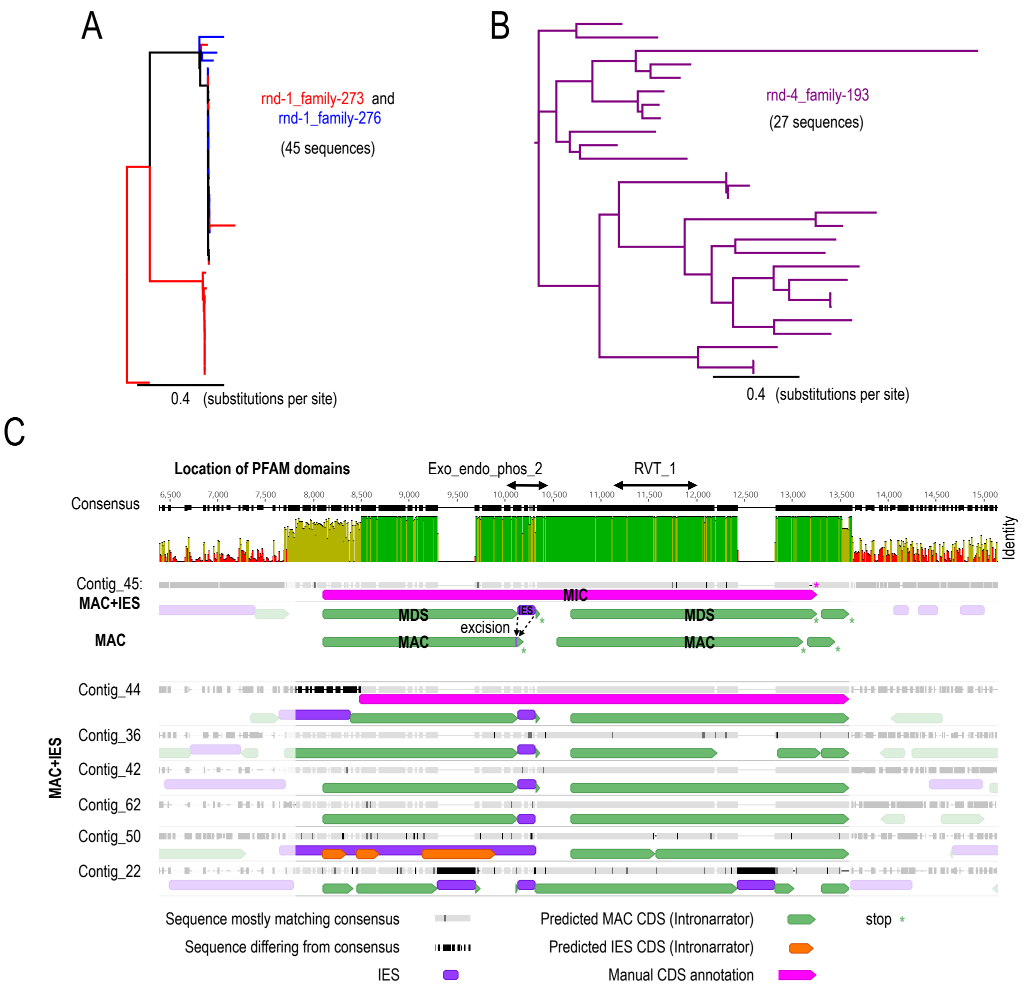

Fig. S7. Non-LTR retrotransposon sequences in both somatic and germline genomes. (*A*) Phylogeny of rnd-1_family-273 and rnd-1_family-276 retrotransposon sequences. (*B*) Phylogeny of rnd-4_family-193 retrotransposon sequences. (*C*) Multiple sequence alignment of non-LTR retrotransposon copies from rnd-1_family-273. Schematic for consequences of IES excision (Contig_45). Identity scale: green=100%; gold=30-99.9%; red=0-29.9%. See also Figure S8.

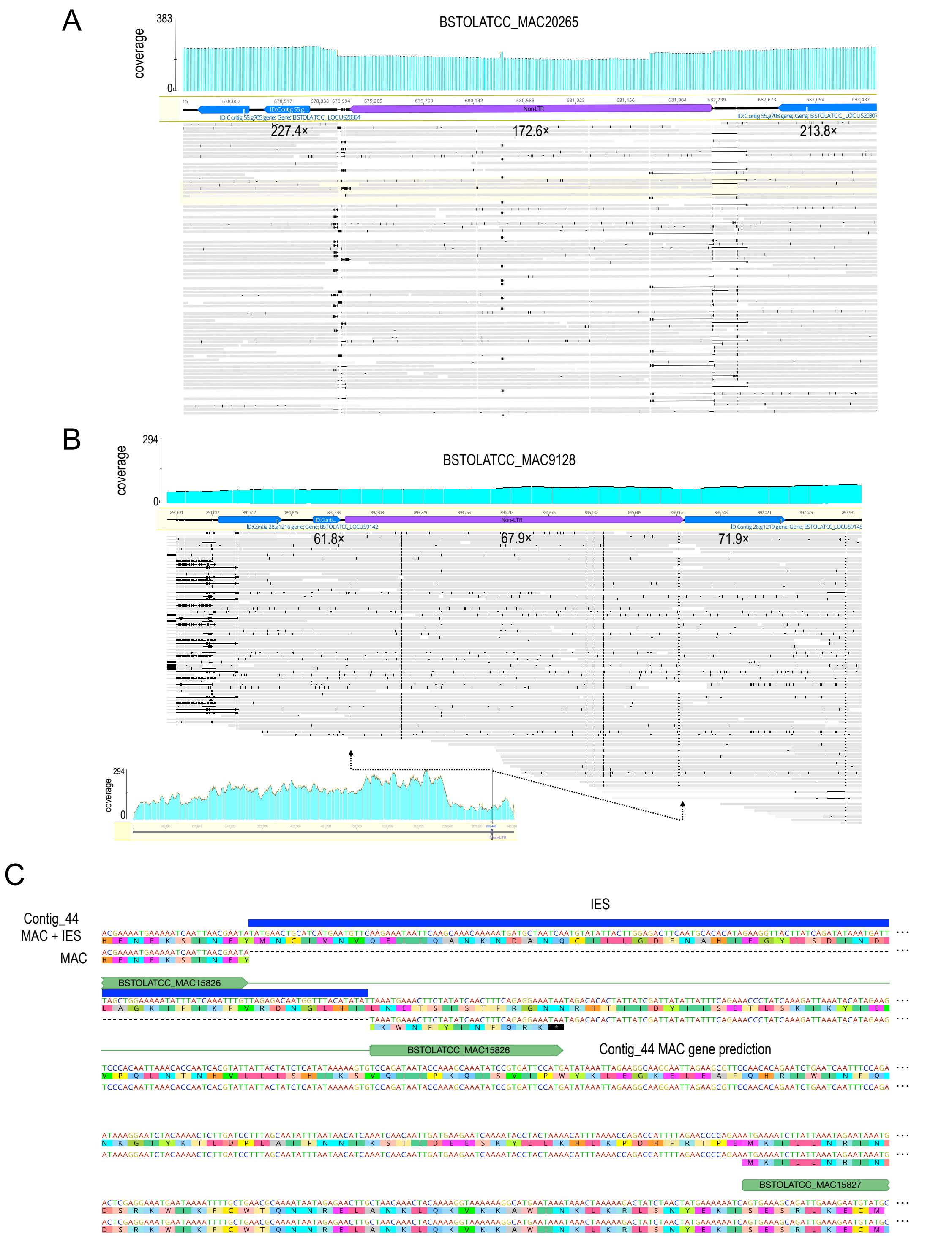

Fig. S8. Non-LTR retrotransposon sequences in both somatic and germline genomes. (*A*) As in Fig. S7C. (*B*) As in Fig. S7C. Inset shows coverage across the entire contig and position of the retrotransposon gene. (*C*) Alignment of MAC+IES and somatic genomic sequences for Contig_44 retroelement genes from Fig. S7D, showing how excision of the central IES deletes part of the endonuclease domain and produces a premature stop codon.

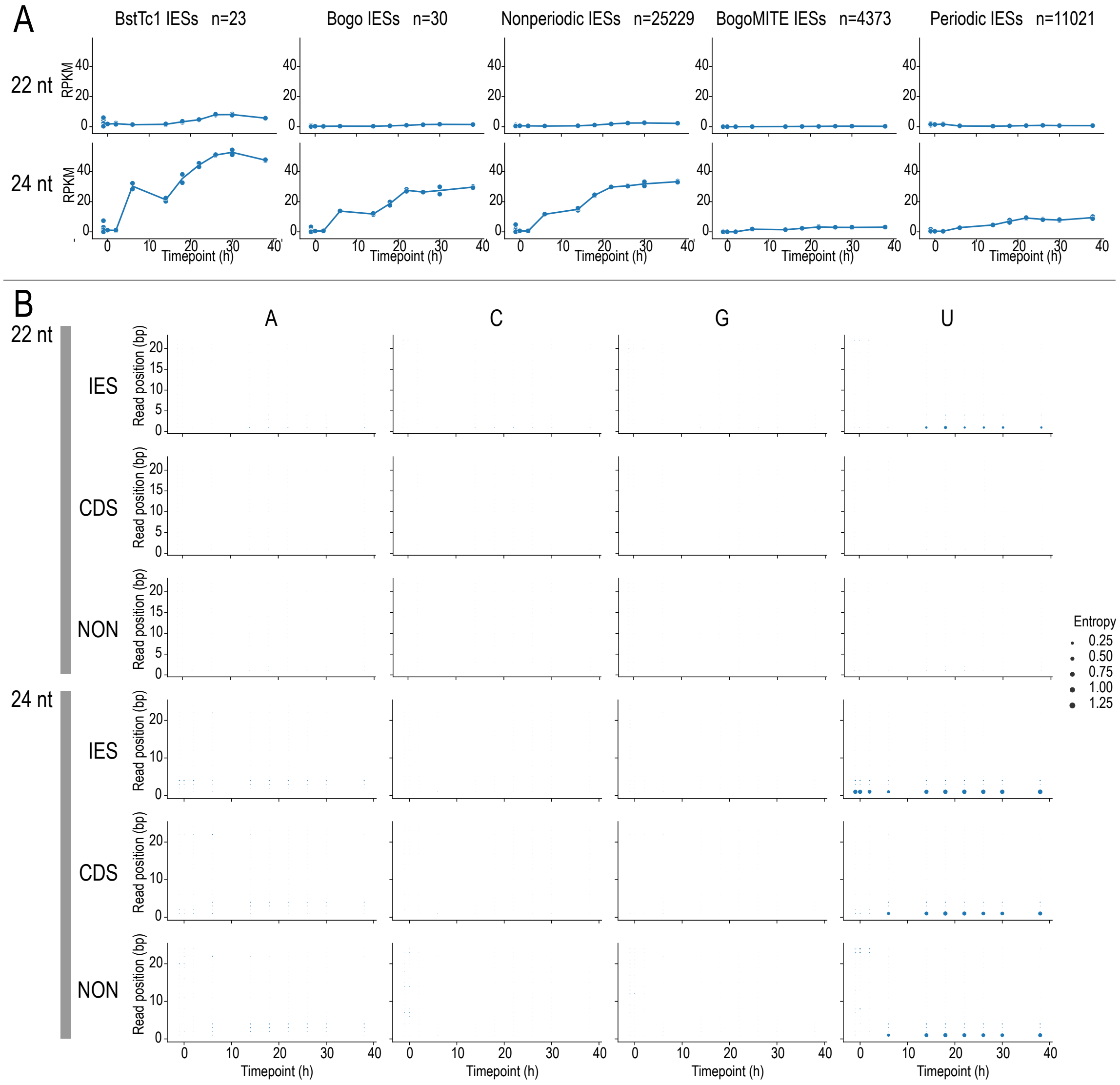

Fig. S9. Differential coverage of development-specific 24 nt small RNAs over different genome features and IES types. (*A*) Relative expression of 22 and 24 nt sRNAs mapping to different categories of IESs: containing full-length copies of BstTc1 and Bogo transposons, at least 90% covered by BogoMITE elements, IESs in the periodic length range (< 115 bp), and all other IESs ("non-periodic"). (*B*) Per-position base entropy of 22 nt and 24 nt sRNAs from developmental time series. Plots show conservation of 5'-U in 24 nt sRNAs. Each plot symbol represents positional sequence entropy (symbol size) for a given nucleotide base (columns) and position in the sRNA sequence (vertical axis) and time point (horizontal axis), in sRNAs mapping to different feature types (rows).

Table S1. IES size classes, defined by peak calling on the length distribution of TA-bound IESs. Lower and upper lengths per size class are inclusive. Only TA-bound IESs on main assembly contigs are included in the counts and total lengths.

Peak center (bp)	Lower bound (bp)	Upper bound (bp)	No. IESs	Total IES length (bp)
65	64	66	207	13415
72	70	74	2717	195724
82	80	84	1035	85017
92	90	94	819	75458
101	99	103	688	69638
110	108	112	1592	175060
153	151	155	336	51478
174	173	175	377	65575
228	225	231	769	175422
389	385	393	876	340798

Table S2. Summary of RepeatMasker annotations in *B. stoltei* MAC+IES assembly for each repeat class, as classified by RepeatClassifier. The most abundant repeat family (rnd-1_family-0) is also listed separately, despite being unclassified. Only one family, rnd-1_family-1, is classified as DNA/TcMar-Tc2. Total annotated length does not account for overlapping annotations.

Class	Number of annotated elements	Total sequence length annotated (bp)
Unknown (excluding rnd-1_family-0)	41836	11279760
rnd-1_family-0 (Unknown)	8369	2692873
Simple_repeat	6878	613736
Low_complexity	2511	123672
rnd-1_family-1 (DNA/TcMar-Tc2)	539	104263
LINE/RTE-X	94	51679
LTR/Pao	39	10475
LINE	28	38630
DNA/TcMar-Tc1	24	38070
Unknown/Helitron-2	23	11025

Table S3. Top five most abundant repeat families in specific IES size classes (defined in Table S1). Repeats comprising > 20% of the total IES length of particular size classes are highlighted in bold font.

Repeat family	Number	Fraction of total IES length	IES size class (peak center bp)
rnd-1_family-397	712	0.044433	65
A-rich	134	0.008362	65
rnd-1_family-157	67	0.004181	65
rnd-1_family-151	65	0.004056	65
rnd-4_family-596	64	0.003994	65
A-rich	2735	0.012229	72
rnd-1_family-438	1898	0.008487	72
rnd-1_family-397	1511	0.006756	72
rnd-1_family-398	508	0.002271	72
(T)n	450	0.002012	72
A-rich	741	0.007556	82
rnd-1_family-397	441	0.004497	82
rnd-2_family-94	182	0.001856	82
rnd-1_family-0	171	0.001744	82
(AT)n	153	0.001560	82
A-rich	570	0.006505	92
rnd-1_family-205	400	0.004565	92
rnd-1_family-0	270	0.003081	92
rnd-2_family-11	209	0.002385	92
rnd-3_family-853	160	0.001826	92
A-rich	801	0.009991	101
rnd-3_family-853	679	0.008469	101
rnd-4_family-1308	277	0.003455	101
rnd-1_family-0	174	0.002170	101

(TATAA)n	128	0.001596	101
rnd-3_family-853	2275	0.011457	110
A-rich	1134	0.005711	110
rnd-2_family-11	336	0.001692	110
rnd-2_family-94	247	0.001244	110
rnd-1_family-210	239	0.001204	110
**rnd-1_family-87**	**14889**	**0.236551**	**153**
rnd-1_family-203	5865	0.093181	153
rnd-1_family-181	1331	0.021146	153
rnd-1_family-93	1059	0.016825	153
rnd-4_family-669	621	0.009866	153
**rnd-1_family-82**	**19793**	**0.268358**	**174**
rnd-1_family-80	6065	0.082231	174
rnd-1_family-93	4335	0.058775	174
rnd-1_family-65	3970	0.053826	174
rnd-1_family-224	2951	0.040010	174
rnd-1_family-160	18889	0.093361	228
rnd-1_family-10	10109	0.049965	228
rnd-1_family-16	9541	0.047158	228
rnd-1_family-14	9344	0.046184	228
rnd-4_family-669	9054	0.044750	228
**rnd-1_family-0**	**294091**	**0.684765**	**389**
rnd-4_family-95	38821	0.090391	389
rnd-3_family-190	9012	0.020984	389
rnd-4_family-1308	5945	0.013842	389
rnd-1_family-25	4430	0.010315	389

Table S4. Numbers of transposase-related Pfam domains in MAC vs. MIC-limited sequences (IESs) for different ciliate species, based on hmmscan search of six-frame translations (6ft), six-frame translations split on stop codons (6ft split, shown in Fig. 4E), or predicted coding sequences only (cds).

	Blepharisma stoltei				*Paramecium tetraurelia*				*Tetrahymena thermophila*				*Oxytricha trifallax*			
	MAC			MIC	MAC			MIC	MAC			MIC	MAC			MIC
Domain	6ft split	6ft	cds	ies 6ft	6ft split	6ft	cds	ies 6ft	6ft split	6ft	cds	ies 6ft	6ft split	6ft	cds	ies 6ft
DDE_1	1	0	3	14	0	0	0	0	0	0	1	83	0	0	0	0
DDE_3	2	2	6	15	1	3	0	7	0	0	3	868	0	0	2	451
DDE_Tnp_1_7	7	0	9	5	1	0	9	0	3	0	3	42	0	0	0	0
DDE_Tnp_IS1595	2	3	5	3	0	0	0	0	0	0	1	138	1	7	7	28
Exo_endo_phos_2	5	0	12	1	0	0	1	0	0	0	1	5	0	0	0	1
HTH_Tnp_Tc5	1	1	4	22	5	9	12	3	0	1	1	56	0	1	2	1
MULE	3	2	6	7	0	0	0	0	0	0	0	2	2	8	6	0
RVT_1	10	4	27	8	0	0	3	4	0	0	3	38	0	0	0	45
Transposase_mut	0	0	0	1	0	0	0	0	0	0	0	0	2	0	1	0
Dimer_Tnp_hAT	0	0	0	0	0	0	0	0	0	3	1	136	0	0	0	0
HTH_Tnp_1	0	0	1	0	0	0	0	0	0	0	0	2	0	0	0	0
HTH_Tnp_Tc3_2	0	0	0	0	0	0	0	0	0	0	0	124	0	0	0	0
Transposase_1	0	0	0	0	0	0	0	0	0	0	0	30	0	0	0	0
Helitron_like_N	0	0	0	0	0	0	0	0	0	0	0	0	0	0	0	3
Tnp_zf-ribbon_2	0	0	0	0	0	0	0	0	0	0	1	0	0	0	0	0
DDE_Tnp_1	0	0	0	0	0	0	0	0	0	0	0	0	0	3	2	0
DDE_Tnp_1_2	0	0	0	0	0	0	0	0	0	0	0	0	0	1	1	0

DDE_Tnp_1_3	0	0	0	0	0	0	0	0	0	0	0	0	0	0	1	0
DDE_5	0	0	0	0	0	0	0	0	0	0	0	0	0	2	0	0

Table S5. Summary of RepeatMasker annotations for individual repeat families that were classified by RepeatClassifier. Repeats identified predominantly in IESs are highlighted in bold. RepeatClassifier classifications that appear to be errors or spurious annotations are surrounded in parentheses: family rnd-1_family-283 mostly comprises ubiquitin sequences, whereas rnd-4_family-1389 contains abundant WD40 repeats.

			All copies				Full length copies only			
**Repeat family**	**Class (Repeat-Classifier)**	**Cons len. (bp)**	**No.**	**Median copy len. (bp)**	**Total len. (bp)**	**No. on IESs**	**No.**	**Total len. (bp)**	**No. on IESs**	**% div. vs. cons**
**rnd-1_family-1**	**TcMar/Tc2**	**1833**	**539**	**91**	**104802**	**505**	**30**	**54844**	**30**	**0.5**
**rnd-1_family-73**	**DNA/TcMar-Tc1**	**1949**	**28**	**1640**	**38098**	**27**	**22**	**36273**	**22**	**0.6**
rnd-1_family-273	LINE	3618	23	1319	38653	2	6	21708	0	16.9
rnd-1_family-276	LINE/RTE-X	3270	15	723	16197	4	2	6451	1	2.95
rnd-1_family-283	(LTR/Pao)	358	39	339	10514	3	24	8438	0	16.25
rnd-4_family-193	LINE/RTE-X	4628	79	279	35576	36	1	4628	1	9.5
rnd-4_family-1389	(Unknown/Helitron-2)	2108	24	268	11049	0	1	2108	0	5.8

Table S6. Counts of intra- vs. intergenic localization for IESs in different size classes (defined in Table S1).

IES size class (peak center bp)	Intergenic	Intragenic	IES size class type	Ratio intra:inter - genic	Fraction pseudo-replicates with higher ratio
65	78	178	periodic	2.282051	0.434
72	851	2300	periodic	2.702703	1.000
82	352	883	periodic	2.508523	0.895
92	331	652	periodic	1.969789	0.013
101	264	549	periodic	2.079545	0.069
110	512	1324	periodic	2.585938	0.995
153	83	199	nonperiodic	2.397590	0.615
174	143	295	nonperiodic	2.062937	0.137
228	257	652	nonperiodic	2.536965	0.913
389	373	767	nonperiodic	2.056300	0.040

SI References

1. C. Denby Wilkes, O. Arnaiz, L. Sperling, ParTIES: a toolbox for *Paramecium* interspersed DNA elimination studies. *Bioinformatics* **32**, 599–601 (2016).
2. M. Singh, *et al.*, Genome editing excisase origins illuminated by somatic genome of *Blepharisma*. *BioRxiv* (2021) https:/doi.org/10.1101/2021.12.14.471607.
3. E. C. Swart, *et al.*, Genome-wide analysis of genetic and epigenetic control of programmed DNA deletion. *Nucleic Acids Res.* **42**, 8970–8983 (2014).
4. L. Duret, *et al.*, Analysis of sequence variability in the macronuclear DNA of *Paramecium tetraurelia*: a somatic view of the germline. *Genome Res.* **18**, 585–596 (2008).
5. O. Arnaiz, *et al.*, The *Paramecium* germline genome provides a niche for intragenic parasitic DNA: evolutionary dynamics of internal eliminated sequences. *PLoS Genet.* **8**, e1002984 (2012).
6. J. S. Fillingham, *et al.*, A non-long terminal repeat retrotransposon family is restricted to the germ line micronucleus of the ciliated protozoan *Tetrahymena thermophila*. *Eukaryotic Cell* **3**, 157–169 (2004).
7. X. Chen, *et al.*, The architecture of a scrambled genome reveals massive levels of genomic rearrangement during development. *Cell* **158**, 1187–1198 (2014).
8. U. E. Schoeberl, H. M. Kurth, T. Noto, K. Mochizuki, Biased transcription and selective degradation of small RNAs shape the pattern of DNA elimination in *Tetrahymena. Genes Dev.* **26**, 1729–1742 (2012).
9. K. Mochizuki, H. M. Kurth, Loading and pre-loading processes generate a distinct siRNA population in *Tetrahymena*. *Biochem. Biophys. Res. Commun.* **436**, 497–502 (2013).
10. C. Hoehener, I. Hug, M. Nowacki, Dicer-like Enzymes with Sequence Cleavage Preferences. *Cell* **173**, 234-247.e7 (2018).
11. A. Prjibelski, D. Antipov, D. Meleshko, A. Lapidus, A. Korobeynikov, Using SPAdes de novo assembler. *Curr. Protoc. Bioinformatics* **70**, e102 (2020).
12. D. R. Zerbino, E. Birney, Velvet: algorithms for de novo short read assembly using de Bruijn graphs. *Genome Res.* **18**, 821–829 (2008).
13. R. C. Edgar, Search and clustering orders of magnitude faster than BLAST. *Bioinformatics* **26**, 2460–2461 (2010).
14. T. Rognes, T. Flouri, B. Nichols, C. Quince, F. Mahé, VSEARCH: a versatile open source tool for metagenomics. *PeerJ* **4**, e2584 (2016).
15. P. Virtanen, *et al.*, SciPy 1.0: fundamental algorithms for scientific computing in Python. *Nat. Methods* **17**, 261–272 (2020).
16. A. R. Quinlan, I. M. Hall, BEDTools: a flexible suite of utilities for comparing genomic features. *Bioinformatics* **26**, 841–842 (2010).
17. R. K. Dale, B. S. Pedersen, A. R. Quinlan, Pybedtools: a flexible Python library for manipulating genomic datasets and annotations. *Bioinformatics* **27**, 3423–3424 (2011).
18. D. Kim, J. M. Paggi, C. Park, C. Bennett, S. L. Salzberg, Graph-based genome alignment and genotyping with HISAT2 and HISAT-genotype. *Nat. Biotechnol.* **37**, 907–915 (2019).
19. M. Stanke, S. Waack, Gene prediction with a hidden Markov model and a new intron submodel. *Bioinformatics* **19 Suppl 2**, ii215-25 (2003).
20. P. Jones, *et al.*, InterProScan 5: genome-scale protein function classification. *Bioinformatics* **30**, 1236–1240 (2014).
21. S. R. Eddy, Accelerated profile HMM searches. *PLoS Comput. Biol.* **7**, e1002195 (2011).
22. J. Mistry, *et al.*, Pfam: The protein families database in 2021. *Nucleic Acids Res.* **49**, D412–D419 (2021).
23. G. Benson, Tandem repeats finder: a program to analyze DNA sequences. *Nucleic Acids Res.* **27**, 573–580 (1999).
24. Z. Bao, S. R. Eddy, Automated de novo identification of repeat sequence families in sequenced genomes. *Genome Res.* **12**, 1269–1276 (2002).
25. A. L. Price, N. C. Jones, P. A. Pevzner, De novo identification of repeat families in large genomes. *Bioinformatics* **21 Suppl 1**, i351-8 (2005).
26. K. Katoh, D. M. Standley, MAFFT multiple sequence alignment software version 7: improvements in performance and usability. *Mol. Biol. Evol.* **30**, 772–780 (2013).

27. J. Storer, R. Hubley, J. Rosen, T. J. Wheeler, A. F. Smit, The Dfam community resource of transposable element families, sequence models, and genome annotations. *Mob. DNA* **12**, 2 (2021).
28. Y.-W. Yuan, S. R. Wessler, The catalytic domain of all eukaryotic cut-and-paste transposase superfamilies. *Proc Natl Acad Sci USA* **108**, 7884–7889 (2011).
29. M. Dupeyron, T. Baril, C. Bass, A. Hayward, Phylogenetic analysis of the Tc1/mariner superfamily reveals the unexplored diversity of pogo-like elements. *Mob. DNA* **11**, 21 (2020).
30. F. Guérin, *et al.*, Flow cytometry sorting of nuclei enables the first global characterization of *Paramecium* germline DNA and transposable elements. *BMC Genomics* **18**, 327 (2017).
31. M. N. Price, P. S. Dehal, A. P. Arkin, FastTree 2 — approximately maximum-likelihood trees for large alignments. *PLoS ONE* **5**, e9490 (2010).
32. D. H. Huson, C. Scornavacca, Dendroscope 3: an interactive tool for rooted phylogenetic trees and networks. *Syst. Biol.* **61**, 1061–1067 (2012).
